# Eye movements and mental imagery during reading of literary texts with different narrative styles

**DOI:** 10.16910/jemr.13.3.3

**Published:** 2020-03-30

**Authors:** Lilla Magyari, Anne Mangen, Anežka Kuzmičová, Arthur M. Jacobs, Jana Lüdtke

**Affiliations:** Hungarian Academy of Sciences, Eötvös Loránd University of Sciences, Hungary; Norwegian Reading Centre, University of Stavanger, Norway; Faculty of Arts, Charles University, Czech Academy of Sciences, Czech Republic; Center for Cognitive Neuroscience Berlin (CCNB) Freie Universität Berlin, Germany; Freie Universität Berlin, Germany

**Keywords:** reading, art perception, eye movement, region of interest, empirical study of literature, mental imagery, reading speed, average fixation duration

## Abstract

Based on Kuzmičová’s [1] phenomenological typology of narrative styles, we studied the specific contributions of mental imagery to literary reading experience and to reading behavior by combining questionnaires with eye-tracking methodology. Specifically, we focused on the two main categories in Kuzmičová’s [1] typology, i.e., texts dominated by an “enactive” style, and texts dominated by a “descriptive” style. “Enactive” style texts render characters interacting with their environment, and “descriptive” style texts render environments dissociated from human action. The quantitative analyses of word category distributions of two dominantly enactive and two dominantly descriptive texts indicated significant differences especially in the number of verbs, with more verbs in enactment compared to descriptive texts. In a second study, participants read two texts (one theoretically cueing descriptive imagery, the other cueing enactment imagery) while their eye movements were recorded. After reading, participants completed questionnaires assessing aspects of the reading experience generally, as well as their text-elicited mental imagery specifically. Results show that readers experienced more difficulties conjuring up mental images during reading descriptive style texts and that longer fixation duration on words were associated with enactive style text. We propose that enactive style involves more imagery processes which can be reflected in eye movement behavior.

## Introduction

Whatever genre of fiction we happen to favour, one of the things that make us prefer a given author over another is their way of expressing everyday sensory experience. Invocations of what it is like to experience the world through our senses have always been an important part of the creative writer toolkit. When such devices succeed in eliciting mental imagery, they can become a source of pleasure to readers [2, 3] as well as an aid to immersion [4, 5], memory for text [2, 6], or even deeper insight [7, 8]. According to some phenomenological theories, “having a rich, immersed reading experience requires active presentification (perception) of the fictional world” [9, p.177]. *Concretization* and *filling-out*, as well as *motor enactment* are assumed to be central to this [10, 11].

There is a long tradition of research into the effects of imageability in the processing of isolated words, e.g., with regard to processing speed [12, 13] or brain activity [14]. However, little empirical research has looked into what makes a natural string of words, such as a fictional narrative, effective in terms of the mental imagery and simulation it elicits (for recent exceptions, see [15, 16]). While single word imageability and concreteness [6, 17] will likely be a contributing factor, the imageability of a longer piece of narrative will always depend on additional text characteristics at higher compositional levels. After all, any literature teacher can speak to how “overly descriptive” story passages easily prove tedious even to the most advanced readers (e.g., [18]). This suggests that simply amassing imageable words may not be enough in producing highly imageable prose.

Our multi-method study integrated qualitative and quantitative text analysis, rating scales and eye tracking in order to explore the relationships between narrative text structure, imageability, and reading experience.

### The role of bodily movement in imagery

In recent decades, several theorists have formulated predictions concerning imagery-prompting features in narrative fiction [1, 10, 11, 19, 20, 21, 22, 23]. Albeit formulated largely independently, these predictions partly overlap. For example, multiple authors [1, 10, 11, 21, 23] propose that in the process of making a storyworld come to life through imagery, verbal renditions of bodily movement play a distinctly productive role. To date, this idea has been most comprehensively developed in Kuzmičová’s [1, 23] theoretical model of mental imagery in narrative reading. Drawing on the embodied language processing [24] and enactivist [25] paradigms in the cognitive sciences, the model predicts that the most strongly imageable prose renders physical objects by way of letting them be manipulated through characters’ bodily movements and actions, rather than explicitly detailing their visual properties as commonly presupposed [26, 27].

Kuzmičová’s [1] model draws a basic distinction between verbal imagery, i.e., auditory or auditory-motor imagery of the wording of a text as if uttered in speech, and referential imagery, i.e., imagery in any sensory modality relating to the contents of the text. The empirical study presented here will focus on referential imagery. It is defined as sensorimotor simulation that has temporarily become *conscious*, i.e., noticeable to the reader. This explicit conceptualization of mental imagery as a type of conscious experience sets Kuzmičová’s [1] model apart from other research on embodied text processing, where mental imagery tends to be conflated with the subpersonal process of sensorimotor simulation as observable in brain imaging and behavioural designs [16, 22, 66, 67]. Unlike sensorimotor simulation, mental imagery thus defined [1] is cognitively taxing. It is also likely to reflect, in any given moment, only some of the sensory content simultaneously activated in sensorimotor simulation (see also [65]).

However, the model suggests that when we read narratives, different sensory modalities can integrate in our imagery in a manner similar to non-conscious embodied simulation as confirmed in the processing of isolated sentences (e.g.[68, 69, 70]). Specifically, this means that mental imagery does not occur with every word of sensorimotor content, but only at distinct “uniqueness points” [69]. On sentence level, an example of such a uniqueness point would be the final noun, “door”, in the sentence “He opened the door.” [69, p. 17]. As the reader processes the verb “opened”, they do not know yet which kind of bodily movement to simulate or imagine, since the verb could be followed by many different expressions suggesting different kinds of actions, including ones that are metaphorical (opening another person’s eyes) or highly complex and extended in time (opening a bank account). The final noun disambiguates the situation and allows an embodied image of the action to be conjured.

Kuzmičová [11, 23] proposes that on the macro levels of groups of sentences, paragraphs, pages, or even chapters in fictional stories, references to object-directed bodily movement or other body–storyworld interactions can do for mental imagery what the word “door” does in the micro example above. Due to the first-person perspective inherent in object-directed action (see also [71], especially when the action is relatively familiar to the reader, the storyworld becomes instantaneously disambiguated in terms of its distance, size, orientation, and so forth relative to the story character’s body. With minimal linguistic means, the reader’s mental imagery is thus provided with ample information regarding the character’s visual focus and their kinaesthetic and tactile sensations, e.g., that of a door handle moving under the pressure of one’s hand (or similar, depending on the type of door that comes to mind).

### The quality of text and imagery: Enactment vs. description

The mental imagery prompted by any piece of narrative can be considered in terms of its “quantity” as experienced by the reader. In this sense, Kuzmičová’s [1] model implies that mental imagery prompted by references to object-directed bodily movement is experienced as stronger than mental imagery prompted by texts that do not feature such references. However, the theory goes beyond modeling quantity in that it distinguishes between two different subtypes of referential mental imagery, forming two ends of a continuum. The first subtype is *enactment-imagery*, wherein the reader instantaneously adopts the inner perspective of a story character as exemplified above. Enactment-imagery can occur in any sensory modality or combination thereof and the suggested prototypical cue of enactment-imagery is a reference to object-directed bodily movement. The second subtype of referential imagery is *description-imagery*, wherein readers do not adopt the perspective of a vicarious experiencer internal to the storyworld, but rather that of a mere visualizer, i.e., someone who is taking in a narrator’s instructions to visually imagine a situation as if from the outside.

Enactment-imagery is proposed to be associated with higher experienced transparency of the linguistic medium and relative ease of mental imaging. Meanwhile, description-imagery is proposed to entail lower transparency of the linguistic medium and thus also a sense of greater effort in conjuring up mental images [1]. The prototypical textual cue of description-imagery, as the term suggests, is static visual description, i.e., such rendition of the storyworld wherein objects are ascribed visual properties but decoupled from characters’ interactions with them. This latter type of cue corresponds more closely to common, intuitive notions of imageable prose [26, 27]. This, however, is not to say that experiences of description-imagery can never be prompted by the cues that are suggested prototypical for enactment-imagery, and *vice versa*.

Kuzmičová’s [1] distinction between descriptive vs. enactment imagery may seem to overlap with other conceptualizations in the field of narrative theory, in particular, with narrative perspective (also called point of view, or focalization [e.g. [28]). Stories may be narrated from a first- or a third-person perspective (or, much more rarely, second-person “you-novel,” as in e.g. Calvino’s *If on a Winter’s Night a Traveler* [1979]). The narrator (whether first- or third-person) may take various positions vis-à-vis the characters, scenes and events in a story, and can have various degrees of access to characters’ inner states (although, restrictions apply for first-person narration in order to retain verisimilitude).

The relationship between immersion, narrative perspective and mental imagery is complex, and few empirical studies have pursued the topic in any systematic manner. A recent exception is Hartung, Burke, Hagoort, and Willems [29], who examined the effect of personal pronoun (first- vs. third-person) referring to the main character (protagonist) on aspects of readers’ engagement with narrative stories. Participants read stories that were written with either first- or third-person pronoun viewpoint. In line with the predictions, results showed that stories with a first-person protagonist led to higher immersion. In particular, participants reported a stronger sense of transportation and higher level of mental imagery after having read the first-person narratives compared to the third-person narratives. It could be assumed, that the observed effects for transportation and mental imagery are not only directly related to the usage of first vs. third person pronouns but mediated by the perspective the reader adapted in her/his situation model. In a subsequent fMRI-study Hartung and colleagues demonstrated that not all readers adopted a first -person perspective when reading a story with first person pronouns [30]. So other variables like the reader’s (situational) preference or additional contextual viewpoint markers like type of verbs used in the story are also important [cf. 31].

In light of Hartung et al.’s [29] findings, it could be assumed that Kuzmičová’s [1] descriptive imagery correspond to third-person narration, and enactment imagery to first-person narration especially when taking into account that enactment imagery is assumed to be accosiated with higher immersion than descriptive imagery. But that is not the case. The key distinction in Kuzmičová’s [1] mental imagery model is not related to narrative perspective, but to whether and to what extent the narrative invites multimodal, embodied engagement and object-directed bodily movement as enacted by the character(s). This means that, even in one text written in one perspective it might be possible to identify passages dominated by enactment imagery and passages dominated by descriptive imagery.

The present study was designed to test the validity of Kuzmičová’s predictions concerning the two distinct types of imagery experience, their grounding in narrative styles featuring the proposed prototypical cues and their role in the reading process and experience.

### Eye tracking research on literary reading

There is a vast literature on what eye tracking data reveals about the reading process [32, 33, 34], and the technology is frequently combined with other behavioural (e.g. [35] and, albeit less often, neuroimaging (e.g.[36, 37]) measures to shed further light on the mechanisms of reading at a fine-grained level. The stimuli in eye tracking experiments tend to be short texts, typically words or sentences, which are often composed and manipulated in light of the hypotheses of the particular experiment. By using lengthy excerpts from existing literary texts, the present experiment aims to contribute to the small but growing area of eye tracking research using more “natural” textual materials such as literary prose or poetry.

The number of eye tracking experiments using literary texts is small, but increasing. Some of these experiments have used different types of poems such as haiku [38] or Shakespearean sonnets [39, 40], whereas others have used short stories [16, 41, 42] or excerpts from novels [43, 44]. Rightly pointing out that no one has ever systematically collected and analyzed the eye movements of participants reading an entire book, Cop, Dirix, Drieghe, and Duyck [45] had monolingual (English) and bilingual (English-Dutch) participants (N=33) read an entire Agatha Christie novel (*The Mysterious Affair at Styles* [1920]) while their eye movements were tracked. Monolingual participants read the novel in the English original, whereas the bilinguals read the first half of the novel in their native language, and the other half in their second language. The data were used to develop the Ghent Eye-Tracking Corpus (GECO), the first bilingual database of eye movements.

Of the extant eye tracking studies using literary stimuli, Mak and Willems [16] is the one most closely related to the present study. Combining eye tracking and questionnaire data, these authors explored the effects of what they term *mental simulation*, rather than imagery, on reading behaviour. Specifically, the aim was to disentangle three types: perceptual, motor, and mental simulation. Perceptual and motor simulation – also called sensorimotor simulation – is elicited by textual descriptions of perceptual and motor events, respectively, whereas mental simulation – the simulation of introspective states, or mentalizing – is elicited by “explicit descriptions of the thoughts, feelings and opinions of a character [and/or] reflection by a character on his own or someone else’s thoughts, feelings or behavior.” [16, p. 514]. Based on the assumption that mental simulation is a time-sensitive process, it was predicted that mental simulation would increase gaze duration, hence slowing down reading speed, in contrast to sensorimotor simulation which would reduce gaze duration. The findings partly supported these predictions, in that not only mentalizing but also perceptual simulation were associated with longer gaze duration and slower reading, whereas textual passages eliciting motor simulation were read faster. Moreover, there were considerable individual differences in the effect of simulation on gaze duration, which were related to individual differences in aspects of self-reported absorption and appreciation [16].

Applying a more theoretically and conceptually fine-grained typology, the present study distinguishes a different pair of readers’ vicarious experiencing, i.e., enactment- imagery and description-imagery. Rather than distinguishing perceptual and motor simulation and mentalizing, we take into account the reader/imager’s notional stance/perspective vis-à-vis the object/action/situation described [23], as guided by salient textual cues. Enactment-imagery, which would roughly correspond to sensorimotor simulation, but also partially to mentalizing in Mak and Willems’ [16] study, is defined as multimodal sensory imagery experienced from an *inner* stance and assumed to be linked with textual cues towards characters’ physical interactions with objects in the storyworld. Meanwhile, Mak and Willems [16, p. 4] operationalize their motor simulation, for example, more simply as simulation of “concrete acts or actions performed by a person or object”. Descriptive imagery, on the other hand, could be related to perceptual simulation and to conscious visual imagery processes of the reader. It does not entail the inner stance of the character or the narrator, and it is assumedly mediated by textual cues such as static visual descriptions of objects.

In our study, we were interested in how Kuzmičová’s [1] typology can be related to the processing of literary text. For this, we used the framework of the Neurocognitive Poetics Model of literary reading (NCPM) developed by Jacobs [46, 47]. Integrating research on the neural, experiential and behavioural processes of reading with research in textual/literary poetics, stylistics and rhetoric, the NCPM hypothesizes a dual route processing of textual material with literary and poetic features: the immersive route and the aesthetic route. All texts vary with respect to their ratio of backgrounded (BG) and foregrounded (FG) features. The central hypothesis of the NCPM is that BG and FG features activate distinct – albeit partially overlapping – neural networks and cognitive-affective processes, with measurable neural, experiential and behavioural effects [46, 47]. Consisting of high-frequency words and a higher degree of predictability than FG texts, texts high in BG features will activate an automatic, fluent, and fast reading mode, typically resulting in higher immersion and transportation. Processes of situation model and event structure building are smooth and effortless, and a sense of familiarity with textual elements facilitates fiction feelings such as empathy, sympathy and suspense [48]. With respect to eye tracking, reading speed should be faster due to shorter and or fewer fixations. In contrast, texts high in FG features typically prompt a dysfluent and hence slower reading, due to their display of unfamiliar, defamiliarizing, textual elements that capture the readers’ attention. The fast flow of reading is disrupted, fixation duration might increase, and more fixations and refixations should appear, making reading speed slower. This is indicative of a “poetic” or “aesthetic” reading mode and would also correspond to Kuzmičová’s [1] characterization of texts cueing descriptive imagery.

Hence, based on the NCPM, we hypothesized that narrative texts with features prompting dominantly enactment-imagery would also prompt higher immersion and transportation during reading, thus being accompanied by shorter fixation duration and faster overall reading speed. Texts cuing description-imagery, however, were expected to elicit a less fluent reading mode with longer fixation duration and slower overall reading speed.

To test our hypothesis, we used longer excerpts from narrative literary texts which prompted dominantly enactment- or description-imagery, respectively, according to qualitative expert judgments provided for this purpose by Kuzmičová. Kuzmičová also defined parts of each text (words, phrases, sentences) especially related to these two kinds of imagery. Having thus samples of dominantly enactive and descriptive text and within these texts parts which were identified as related to descriptive or enactive imagery, we conducted two studies. In the first study, we tested through quantitative analyses of word category distributions whether the texts and the indicated parts of texts indeed contained different textual cues, as associated with their assigned imagery type. We expected enactive texts and parts of all texts theoretically prompting enactive imagery to contain more verbs and descriptive texts and parts of all texts prompting descriptive imagery to contain more nouns and adjectives (Example 1).

“Later that day, I went up to a shabby café on the coast road to the north of my town where he and a few friends, along with other people I didn’t know, had **come to watch a performance of some kind that included primitive tribal chants. When I came in, the room was already darkened except for the spotlights on the stage.** The only empty chair I could see at the long table was the one next to him, though a piece of clothing and maybe a purse were hanging from the back of it...”

„Später fuhr ich zu einem schäbigen Cafe an der Küstenstraße nördlich der Stadt, in der ich lebte, und er und ein paar Freunde und andere Leute, die ich nicht kannte, waren dorthin gekommen, **um irgendeine Aufführung mit primitiven Stammesgesängen zu sehen. Als ich den Raum betrat, war dieser, abgesehen von der Bühnenbeleuchtung, bereits verdunkelt.** Der einzige freie Stuhl an dem langen Tisch, den ich entdecken konnte, war der neben ihm, obwohl von der Rückenlehne ein Kleidungsstück und vielleicht eine Handtasche hingen...“

**Example 1.** An excerpt from the Descriptive Text stimulus by author Lydia Davis (English original and German translation). Bold letters show text-parts coded for prompting enactment imagery, text-parts with underline is coded to prompt description imagery.

In the second study, participants read the texts in an eye-tracking paradigm. In addition to studying their eye movement behaviour, we studied their reading experience (including their experiences of imagery and feelings of immersion and transportation) using a post-reading questionnaire. In our analysis of eye-movements, we analysed reading speed at page level. Afterwards a more fine-grained analysis was conducted on single word-level focussing on the average fixation duration, first fixation duration, gaze duration, dwell time and number of revisits in those parts of texts that were indicated by Kuzmičová as distinctly prompting enactment- and description-imagery (see Example 1).

## Study I: Analysis of word-categories

### Methods

#### Stimuli

German translations of four texts were selected, combined in pairs of two. One text in each pair was predicted to prompt description-imagery (Descriptive Text) and the other was predicted to prompt enactment-imagery (Enactive Text; based on an ad hoc qualitative coding by Kuzmičová). One text pair consisted of German translations of two texts by different French authors (here called French texts), one by Georges Perec (*Les Choses*, orig. published 1965; German translation *Die Dinge*, translated by Eugen Helmlé, publ. 2004) and one by Jean-Phillippe Toussaint (*L’Appareil-photo*, orig. published 1989; German translation *Der Photoapparat*, translated by Joachim Unseld). Both excerpts were taken from the beginning of the novels without any changes. The other text pair consisted of two excerpts from the German translation of the novel *The End of the Story* by Lydia Davis (orig. published 1995; German translation *Das Ende der Geschichte*, translated by Klaus Hoffer, publ. 2009) (here called Davis texts). Both excerpts were taken from the beginning of the novel (pp. 2-7 for enactment-imagery, and pp. 9-21 for description-imagery). For the purposes of the present study, some parts of the original texts were deleted in order to include more parts of the text that were predicted to be suggestive of either enactive or descriptive narrative style. Care was taken to maintain the coherence and comprehensibility of the texts despite the deletions (see Appendix Stimuli for all texts). It was also indicated by Kuzmičová which parts of the texts (words, phrases, sentences) would be distinctly linked with enactment or description imagery in each text.

The length of the text excerpts was 1274 (Perec Descriptive Text), 1373 (Toussaint Enactive Text), 1181 (Davis Descriptive Text) and 1227 words (Davis Enactive Text).

#### Coding and analysing word-categories

We automatically annotated all texts with part-of-speech using TreeTagger and the Stuttgart-Tübingen-Tagset [49]. After the initial tagging, the several word forms from STTS were aggregated in the broader categories adjectives, adverb, articles, conjunctions, nouns and proper names, numbers, words from other languages, particles, prepositions, pronouns and verbs. We analysed the ratio of three main categories of words, verbs, nouns and adjectives in two levels of the texts. First, we counted the number of the main word categories, verbs, nouns and adjectives in each of the four texts. Then, we compared by chi-squared test within each text-pair (Davis and French texts) for each word class whether there was a higher or lower frequency of any of the three choosen classes in the Enactive Text compared to the Descriptive Text of each text-pair. In our second analysis, we analysed only those text-parts (words, phrases and sentences) which were indicated by Kuzmičová as theoretically prompting description or enactment imagery. We again counted the number of three main word categories, verbs, nouns and adjectives in these text-parts within each text. Then, we compared by chi-squared test within each text-pair for each word category whether the frequency of these categories of words was the same for text parts prompting enactment vs description imagery. In this analysis, we collapsed text-parts across the different (Enactive vs Descriptive) Texts within a text-pair (i.e., we did not take into account to which text within the text-pair a text-part belonged to).

### Results

We compared the ratio of three main word categories, verbs, nouns and adjective in the Enactive and Descriptive Texts within each text-pair (see Table 1). In both text-pairs, there were more verbs in the Enactive compared to the Descriptive Text (Davis text-pair: χ^2^=6.865, p=0.009; French text-pair: χ^2^=30.297, p<0.00001). There was no difference in the number of nouns and adjectives in the two Davis texts (Nouns: χ^2^=1.157, p=0.282; Adjectives: χ^2^=3.152, p=0.076), but there were relatively more nouns and adjectives in the French Descriptive Text compared to the French Enactive Text (Nouns: χ^2^=68.317, p< 0.00001; Adjectives: χ^2^=43.866, p< 0.00001).

**Table 1:** Number of all Words and Number of Words in Word-categories per Texts

Number of words	Descriptive Text	Enactive Text	Chi-square test
Davis text-pair			
All	1181	1227	
Verbs	183	240	**
Nouns	209	197	n.s.
Adjectives	107	87	n.s.
French text-pair			
All	1274	1373	
Verbs	147	265	***
Nouns	374	219	***
Adjectives	195	99	***

*Note*. The fourth column shows whether there was a significant difference in the frequency of word categories per text within each text-pair

n.s.: not significant. ** p<0.01. ***p<0.001.

We also checked the ratio of word categories on all parts of texts which were indicated as prompting description or enactment imagery. In the Descriptive Text in the Davis pair, 346 words were marked as descriptive and 141 as enactive, while in the Enactive Text in the Davis pair, 28 words were marked as descriptive and 412 as enactive. In the Davis pair of texts, there was no difference in the ratio of verbs (χ^2^=1.300, p=0.254), nouns (χ^2^=0.196, p=0.658) and adjectives (χ^2^=0.854, p=0.355) in all words marked enactive compared to the words marked descriptive (irrespective of which text the words belonged to). In the French texts, however, there were more verbs among the enactive words compared to the descriptive words (χ^2^=19.5514, p<0.00001), and there were more nouns and adjectives among the descriptive compared to the enactive words (Nouns: χ^2^=12.6295, p<0.0004; Adjectives: χ^2^=36.5161, p<0.00001).

**Table 2:** Number of all Marked Words and Number of Marked Words in Word-categories in Descriptive and Enactive Text-parts

Number of words	Descriptive parts of texts	Enactive parts of texts	Chi-square test
Davis text-pair			
All marked	374	553	
Verbs	50	89	n.s.
Nouns	83	116	n.s.
Adjectives	48	60	n.s.
French text-pair			
All	411	576	
Verbs	32	101	***
Nouns	124	117	***
Adjectives	93	51	***

*Note*. The fourth column shows whether there was a significant difference in the frequency of categories of words per text within each text-pair

n.s.: not significant. ***: p<0.001.

### Interim Summary (Study I)

In this study, we explored whether we can find more textual cues related to description and enactment imagery in texts and parts of texts which were judged by Kuzmičová as prompting these types of imagery. Therefore, we compared the frequency of three main word categories: verbs, nouns and adjectives. We expected Enactive Texts and parts of all texts prompting enactment imagery to contain more verbs and Descriptive Texts and parts of all texts prompting description imagery to contain more nouns and adjectives.

Within the Davis text-pair, we found that the Enactive Text contained more verbs than the Descriptive Text, however, we found no word category differences within the parts of texts which were marked as prompting either enactment or description imagery. Within the French text-pairs, we found differences in the frequency of all three word categories when the Enactive and Descriptive Texts and also when the marked parts of texts were compared. Indeed, there were more verbs and fewer nouns and adjectives in the Enactive Text and text-parts.

Although the Davis texts showed fewer differences in the categories of words, the results for the French texts confirm that the two narrative styles are associated with different categories of words which are potential cues for the different types of imagery. It also seems that differences in the number of verbs might be a stronger cue for enactment vs. description imagery compared to the two other categories of words, because the number of verbs was also different in the Davis text-pair.

## Study II: Reading experience and eye movements

### Methods

#### Participants

Twenty participants (13 females; *M*_age_ = 29.3 years, *SD*_age_ = 8.602, age range: 20-47 years) were recruited from the participant database of the Freie Universität Berlin. Additional 10 participants also participated in the experiment but their data got excluded because of their low quality (e.g., lost signal or poor calibration). All participants were native German speakers, had corrected or corrected-to-normal vision, and were paid ten euros for their participation. They were naive to the purposes of the experiment and were not trained literature scholars. Half of the participants (ten) had a high-school diploma, and additional eight persons also finished their bachelor or their master degree. Seventeen persons had German as their only native language, three participants were multilingual.

#### Ethics statement

The study was conducted in the eye tracking lab of the Department of Education and Psychology, Freie Universität Berlin (FUB). All standard experimental procedures involving the collection of purely behavioural data (in the present study: eye movements, audio recordings, comprehension tests, rating data), not requiring any invasive or potentially harmful methods, are approved by the ethics committee at FUB (ref. 86_2/2014), and all subjects gave informed consent in accordance with the Code of Ethics of the World Medical Association (the Declaration of Helsinki). Data were stored and analyzed anonymously.

#### Stimuli

For this study, we used the same four text excerpts which were analysed in our first study: an Enactive and a Descriptive Text (excerpts) from the German-translation of the same novel by Lydia Davis (Davis text-pair), and other two excerpts of novels translated to German, an Enactive Text written by Jean-Phillippe Toussaint and a Descriptive Text written by Georges Perec (French text-pair). These texts were relatively homogeneous in that they all relate mundane, non-dramatic situations and events, free from overt twists of plot (e.g., a walk through a city, a visit to driving school). Thus, they all make sensory experience stand out as relatively salient (in comparison to, e.g., suspense or symbolic potential), perhaps also to readers who may be generally little inclined to conjure mental imagery. While all the texts originated in linguistic areas foreign to the participants of the study, the quality of the German translations was checked, and none of the excerpts rendered settings that could be assumed too unfamiliar to participants to allow spontaneous imagery.

The texts were 17-20 “pages”/screens long, each screen (“page”) consisting of between 9 and 11 lines, presented with double line space, in a 32 point Arial black font, maximum letter size 0,7 cm. Page numbers were at the bottom of each page. Maximum text height (corresponding to 11 lines) was 22 cm (10-line pages were 20 cm, 9-line pages were 17,8 cm), maximum line width was 21 cm. All pages were aligned so that the first line appeared in the exact same location and, whenever possible, line breaks were set to avoid word-splits. Line numbers slightly differed across pages in order to have sentence endings at the end of pages. However, due to the complexity of the texts, it was impossible to avoid that, in some instances, the final sentence of one page would continue on the next page. The sectioning of the texts (i.e. intended lines) was done in accordance with the authors’ original division into paragraphs.

At the beginning of the experiment, all participants read a shorter text (an excerpt from Thomas Mann’s *Buddenbrooks*), for practice.

#### Apparatus

The stimuli were presented using SMI Experiment Center 3.7.6.0, on a 19 inch Samsung LCD computer screen with 1280x1024 resolution with 86 dpi and a 60 Hz refresh rate, and a viewing distance at approx. 60 cm. The eye movements were recorded with a SMI RED eye tracker set to a sampling rate of 250 Hz. To avoid excessive head movement, we used a chin rest that was adjusted individually for optimal reading posture. Rating data was collected on a separate laptop using SoSci Survey (https://www.soscisurvey.de/ ).

#### Design and Behavioural Measures

In a 2 (Enactive and Descriptive Texts as within-subject variables) by 2 (text-pairs: French (Perec/Toussaint) and Davis as between-subject variables) design, participants read two texts (either the two French or the two Davis texts) on a desktop computer while their eye movements were recorded, and responded to the following questions, in this order: three multiple choice comprehension questions after each text, three open response questions asking specifically about participants’ imagery (not analysed in this study), 18 items assessing aspects of their reading experience of the given text, an 18-item personality questionnaire (administered after participants had read the first text) (not analysed in this study), and demographic information.

The demographic information consisted of general demographic questions (gender, age, education, native language(s)) and questions about the participants’ reading habits. 18 items using a 5-point Likert scale assessed participants’ subjective reading experience of each text (see Appendix Reading experience questionnaire for full list of items with references to the original studies). The reading experience questionnaire combined items developed by us and items from existing questionnaires [50, 51, 52] sometimes with slight modifications. Four items assessed different aspects of mental imagery (Items 1-4 in Table A1). The other items asked about immersion (Item 6), feeling of transportation (Item 7), suspense (Item 8), emotional involvement (Item 9-10), general reading pleasure (Item 11), cognitive involvement (Item 12-13), difficulties with attention during reading (Item 5), awareness of the coherence (Item 14), cognitive access to the content (Item 15-16), atmosphere conveyed by the text (Item 17) and about empathy evoked towards the situation described in the text (Item 18) (see all items in Table A1). The items were presented in random order to participants, and they were always in the same order after each text for the same participant.

#### Procedure

Participants were instructed that they would be reading excerpts from two novels (either the two texts by the two French authors or the two texts by Davis) while their eye movements are recorded, and that they would be asked some questions about their reading experience afterwards. Informed written consent was obtained by all participants after the information about the study and the procedure. The session started with a short practice text so that they could get more familiar with the reading situation (e.g., the feel of the chin rest, how to turn the pages) and answered three comprehension questions. In order not to interrupt the flow of reading, calibration was done only at the beginning of each reading session and not between the pages.

After calibration, the participant read Text 1 at her own pace, turning the page by using the spacebar. Then, she answered three multiple choice questions. After having finished the comprehension questions, the participant notified the experimenter, who then asked few open questions using the microphone and recorder. This was followed by the rating questions and the personality questionnaire, for which the participant moved to another computer in the same room. When finished, the participant went back to the eye tracker to read Text 2, and then the procedure was repeated as for Text 1. Demographic information was collected at the end of the session, and the session finished with a short debriefing, during which we also asked the participants whether they recognized, or had read, any of the texts (all the participants in our sample responded “no” to this question). The order of the texts was counterbalanced between participants. The session lasted around 60 minutes.

#### Preprocessing of eye movement recordings

Fixations were identified based on the standard dispersion based algorithm offered by SMI. Before analysing eye movements, all participants’ data was checked for drifts. One of the most common type of drift was that the fixations were slightly above (especially, at the upper part of a page) or under (at the lower part of the pages) of the lines they belonged to. Therefore, if necessary, fixations were vertically aligned to the line they belonged to in SMI BeGaze.

We conducted three types of analysis of the eye movement data. First, we analysed the reading speed of the pages. For this, we asked our expert, Kuzmičová, to rate the narrative style of each paragraph as descriptive, enactive or none of the two (neutral).

Descriptive paragraphs were paragraphs in which the narrator primarily ascribed visual properties to environments. Human characters, if at all mentioned, did not physically interact with these environments other than simply being “in” them. Enactive paragraphs, on the other hand, were paragraphs in which human characters were given more agency *vis-à-vis* their environment – manipulating, touching or otherwise perceiving it through their inner senses such as taste or smell [1, 11]. There were also paragraphs where the physical environment was not a salient theme.

Then, we selected reading speed of those pages for analysis which contained only either descriptive or enactive paragraphs. Pages which contained both type of paragraphs or did not contain any paragraph with descriptive or enactive style got excluded. In this way, we used 8 descriptive pages and 3 enactive pages from the Davis Descriptive Text, 1 descriptive page and 10 enactive pages from Davis Enactive Text, 13 descriptive pages and no enactive pages from the French Descriptive Text, and 1 descriptive page and 13 enactive pages from the French Enactive Text.

Second, we analysed different duration based measures at word-level. For these analyses, we used only the Davis text-pair. As reported later in the analysis of the reader experience ratings, the self-reported reading experiences of the Enactive and Descriptive Texts of the French text-pair differ from each other not only for imagebility related items but also for other sub-scales like suspense and coherence. We assume that the effect of imagery style on eye movements might be better understood when only the more homogenous Davis text-pair is examined. For the analysis at word level, we therefore included only those words from the Davis texts 1) which belonged to a text-part indicated by our expert as prompting enactive or descriptive imagery [(see Study I), 2] which were verbs, nouns or adjectives and 3) which were fixated. To get a detailed picture we analysed mean fixation duration (mean duration of all fixations on the words satisfying the above mentioned criteria) as a general indicator of reading behaviour, first fixation duration, and gaze duration (sum of fixation durations at the first visit) , two measures associated with early and more automatic processes of lexical processing, and dwell time (sum of all fixations and saccades of all visits on a word), a measure associated with both early and later processes like deeper semantic processing, reanalysis and integration (e.g., [33, 53]). Due to technical reasons, gaze duration was calculated for words with one visit and for words with more visits if the number of fixations were not more than the number of visits +1. Therefore, 92% of the gaze duration was analysed.

Third, we also analysed whether a regression occurred on a word after its first reading (coded as a binary variable). The amount of revisits or regressions are usually interpreted as an indicator for comprehension difficulties (e.g. [53, 54]). Also for this analysis only words from the two Davis texts satisfying the above mentioned criteria were used.

For the analysis of reading speed (calculated as words per minute) and the different measures of fixation duration (measured in ms), we used linear mixed-effect models with maximum likelihood criterion in R (the lmer function in “lme4” package [55]. In the page-level analysis (i.e. reading speed), random factors were the different pages and participants, and the random slope of imagery type of pages per participants was also included (Item: *χ^2^*=239.31, *p*<0.001, Participant: *χ^2^*=546.66, *p*<0.001, Imagery type: *χ^2^*=34.31, *p*<0.001). In the word-level analyses, random factors were the words, participants and the random slope of imagery type of words per participants (for mean fixation duration: Words: *χ^2^*=120.57, *p*<0.001, Participants: *χ^2^*=157.63, *p*<0.001, Imagery type: *χ^2^*=24.528, *p*<0.001; for first fixation duration: Words: *χ^2^*=68.501, *p*<0.001, Participants: *χ^2^*=138.29, *p*<0.001, Imagery type: *χ^2^*=17.017, *p*<0.001; for gaze duration: Words: *χ^2^*=218.42, *p*<0.001, Participants: *χ^2^*=121.37, *p*<0.001; for dwell time: Words: *χ^2^*=234.88, *p*<0.001, Participants: *χ^2^*=394.43, *p*<0.001, Imagery type: *χ^2^*=14.59, *p*<0.001). For gaze duration, the random slope of imagery type did not contribute significantly to the model, therefore, it was not included (Imagery type: *χ^2^*=1.827, *p*=0.401).

To test whether random factors significantly contribute to any model, we compared an “intercept only” model with both random factors and with a similar model without the given factor. We also compared an “intercept only” model with the two random factors included and a similar model including the random slope. The “intercept only” model with both random factors was response variable ~ 1+(1|Participant) +(1|item). We included imagery type as random slope because this was the variable in the focus of our study. We did not include other variables as slopes to avoid models which are not converging.

We used backward model selection starting with a full model which included all predictor variables and also the interaction of text pair and imagery type. Predictor variables were step-wise removed if they did not contribute significantly to the model. First, the interaction term was examined and then any other variable which had the smallest beta value in a given model. If a model failed to converge when one of the variables were removed, the variable with the next smallest beta value was examined. Significance of the variables was evaluated by comparing the models with likelihood-ratio test (using the anova function in R).

For the analysis of revisits, we created a binary variable which showed whether a word was revisited (fixated again) after its first reading or not. We calculated a binomial general linear mixed-effect model using the glmer function from the “lme4” R package with binomial family specification [55]. The structure for the random (Words: *χ^2^*=44.941, *p*<0.001, Participants: *χ^2^*=85.986, *p*<0.001, Imagery type: *χ^2^*=6.299, *p*=0.042) and the fixed effects were the same as for the duration-based measures. We used forward model selection in this analysis because the full model which included all predictor variables did not converge. We examined all predictor variables by including them in the model in a step-wise manner. Only variables with a significant effect remained in the model.

### Results

#### Comprehension of the texts

All participants could correctly answer at least one out of the three comprehension questions per text, on average they correctly answered 2.275 comprehension questions (*min*=1, *max*=3, *mean*=2.275, *sd*=0.784). Wilcoxon signed-rank test showed no difference in the comprehension scores between the two French texts (*median_descriptive_*=2, *median_enactive_*=2.5, *V*=4.5, *p*=.408) and between the two Davis texts (*median_descriptive_*=2, *median_enactive_*=2, *V*=13.5, *p*=.516). There was no difference between the comprehension scores of the two group of participants who read different texts according to the Wilcoxon rank-sum test (*median_French texts_*=2, *median_Davis texts_*=2.5, *W*=208, *p*=.815).

#### Reading experience ratings

We analysed the responses to the reading experience questionnaire to explore differences in the reading experience between the Enactive and Descriptive Texts within the Davis and within French text pairs. We also looked for differences between the two text pairs. When items were part of the same scale (e.g. item 9-10 is from Appel et al.’s (50) Emotional Involvement scale), scores were summed, and the sum score was analysed. When scales were summed, scores for item 12 and 16 were inverted. Although two of the imagery items were also part of the same scale, we analysed all of them separately because all four imagery items tap into different aspects of imagery. Because of the non-normal distribution of all ratings we used the non-parametric Wilcoxon signed-rank test to compare the Enactive and Descriptive Texts and the non-parametric Wilcox rank-sum test to compare scores between text-pairs. P-values were not corrected for multiple comparison. Table 3. shows the results for each item and item-pairs with summed scores.

**Table 3:** Results of the Reading Experience Questionnaire

Item	French		V	p	Davis		V	p	Text-pairs		W	p
	Descr	Enact			Descr	Enact			French	Davis		
1 Imagery	3.5	4	0	**.039**	2.5	4	0	.063	4	4	169.5	.366
2 Imagery	3	4	0	**.017**	3	4	2	.129	3	4	161.5	.274
3 Imagery	3	2	32	**.047**	3	2	26	**.039**	2	2	211.5	.746
4 Imagery	3.5	4	7	.058	4	4	8.5	.340	4	4	196	.908
5 Attentional Focus	3.5	2	23.5	.101	3.5	2.5	8.5	.785	3	3	213	.717
6 Immersion	3	4	0	.065	1.5	3	5	.120	2	4	117	**.019**
7 Spatial Presence	3	3	4	.163	3	3.5	0	.058	3	3	178	.534
8 Suspence	2.5	4	2	**.020**	3	3.5	8	.256	3	3	169.5	.388
9-10 Emotional Involvement	4.5	5.5	6.5	.2	4	7	4.5	.106	4	5	187.5	.731
11 Overall Reading Pleasure	3	3.5	1.5	.055	2	4	2	.068	2.5	3	191	.8
12-13 Cognitive Involvement	3.5	2	1.5	.057	3	2	12.5	.121	2.5	3	174.5	.48
14 Coherence	2.5	4	3	**.018**	2.5	3	2	.256	3	3.5	143	.108
15-16 Ease of Cognitive Access	3	7	0	**.004**	4	7	17.5	.296	7	5	226	.476
17 Atmosphere	2.5	4	0	**.015**	4	4	6	.317	4	4	228	.389
18 Empathy	4	4.5	6.5	.098	2.5	4	1	.140	4	4	136.5	.064

*Notes*. Sum scores were calculated for item 9-10, 12-13 and 15-16. Column 2,3 and 5,6 and 8,9 show the median of the scores on the 5-point Likert-scale. Column 4, 7 and 10 show the V, W and p-values of the Wilcoxon signed-rank and the Wilcoxon rank-sum test.

p < 0.05 are written in bold

**Figure 1:**
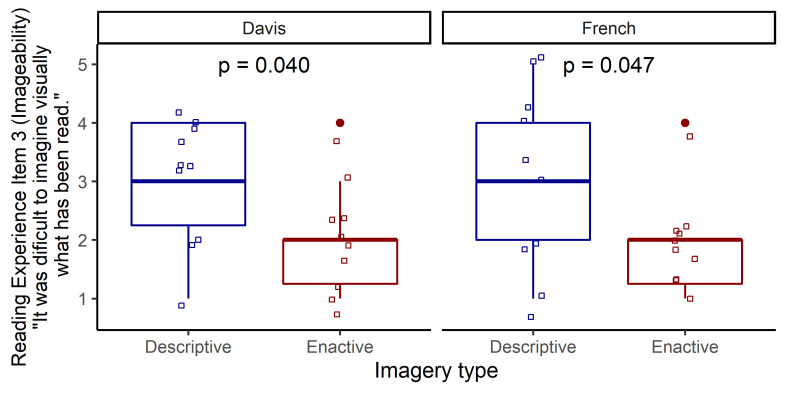
Results for Item 3 (Imageability) of the Reading Experience Questionnaire

*Note*. Wilcoxon singed-rank test showed significant differences between Descriptive (blue) and Enactive (red) texts within both text pairs (Davis (left two boxplots) and French (right two boxplots)).

Within both text pairs, Item 3 which asks about the difficulty of visual imagery was lower for the Enactive Text (Figure 1).

**Figure 2:**
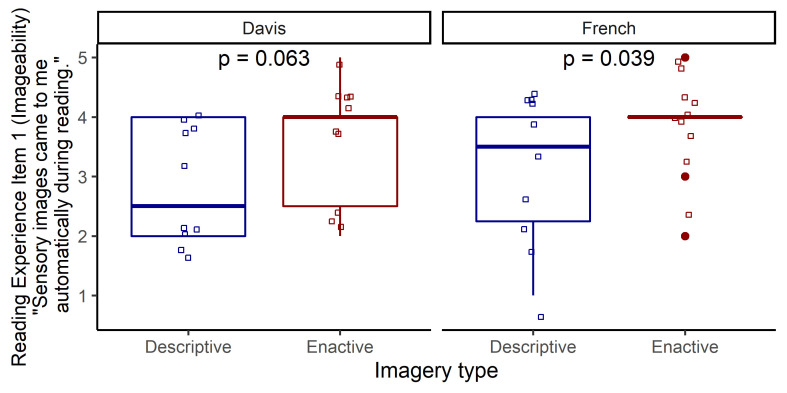
Results for Item 1 (Imageability) of the Reading Experience Questionnaire

*Note*. Wilcoxon singed-rank test showed significant difference between descriptive (blue) and enactive (red) texts within the French text pairs (right two boxplots) and trend within the Davis texts (left two boxplots).

Another imageability item (Item 1) which asks about the automatic appearance of sensory images showed significantly higher scores for the Enactive Text within the French text pair and a trend within the Davis texts (Figure 2).

Additional items, namely Item 2 (Imageability), Item 8 (Suspense), Item 15-16 (Ease of Cognitive Access), 14 (Coherence) and 17 (Atmosphere) got higher scores for the Enactive Text within the French texts but not within the Davis text pair. Scores of one item, Item 6 (Immersion), were higher for the French texts compared to the Davis texts by Wilcox rank-sum test (*median_Davis_*=2, *median_French_*=4, *W*=117, *p*=0.019), but there was no difference between the texts on other items.

To sum up, one of the imageability items (Item 3) showed lower scores for the Enactive texts in both text pairs, indicating that imagery was perceived as more difficult for the Descriptive Texts. There were other items showing differences between Enactive and Descriptive Texts but only within the French text pair. The reading experience of the two French texts seemed to differ not only in imageability but also in suspense, coherence, cognitive access and atmosphere.

#### Eye tracking results

##### Reading speed of pages

We tested which variables affect the reading speed of pages with enactment and description imagery. The response variable, reading speed was the log-transformed value of the number of words per minute for each page and participant (*mean*=2.324, *min*=1.834, *max*=2.683, *sd*=0.133). Outlier values (reading speed values smaller or larger than two times of the standard deviation from mean per participants) were rejected.

For predictor variables, we included some features of the pages which might influence reading speed: 1) the page number which shows how far the participant got in the whole text, 2) the mean of the automated readability index (ARI [56]) of the sentences on each page, 3) number of words on each page, 4) the ratio of verbs on the page compared to all words, 5) the ratio of adjectives on the page compared to all words. ARI is designed to estimate the understandability of texts. Its calculation is based on the number of words and letters in a sentence. We calculated ARI for each sentence and averaged the ARI values of the sentences on a page. The ratio of the different main word classes was included because we found in study I that those might be related to the characteristics of the different types of texts. The ratio of nouns on the pages were not included because it highly correlated with other variables (correlation with ratio of verbs: r=−0.867, p<0.001 and with mean log frequency of words: r=−0.793, p<0.001). We also included variables related to certain features of words on the pages: 6) mean of the log-transformed value of word frequency, 7) mean of the aesthetic potential value of words, 8) mean of the arousal value of words, 9) mean of the imageability of words. These variables were calculated with the help of the German subtitle-based word corpus SUBTLEX-DE [57]. The hitrates/coverage for all four variables calculated based on SUBTLEX-DE corpus were quite high: 85.1% for the French Descriptive Text, 95.5% for the French Enactment Text, 93.7% for the Davis Enactment Text and 95.8% for the for Davis Descriptive Text. It is well estrablished that higher frequency words are read faster than lower frequency words (see e.g. [58]). Aesthetic Potential (AP) is a measure quantifying word beauty. The estimates are based on an algorithm calculating the semantic relatedness of a target word with a list of affective-aesthetically positive and negative words [59]. The AP feature is highly correlated to valence and liking ratings [60], and it is associated with aesthetic appreciation [59]. Imageability is computed based on the algorithm reported in Westbury et al. [13] using the size and density of a word’s semantic context and the emotional associations of the word. Imageability has been related to behavioural effects in lexical decision task [13]. Similarly to AP, arousal is also computed with an algorithm based on semantic associations of a target word with a list of high and low arousing key words [60]. The lexical semantic features, arousal and imageability induced higher immersion ratings for example, in Jacobs and Lüdtke’s study [48] while participants read a short story. We also added categorical variables related to our experimental manipulation: 10) text pair (whether the page belonged to the French or to the Davis texts) and 11) imagery type of the pages (enactive or descriptive). All predictor variables were standardized. Interaction between imagery type and text-pair was also included in the starting model. The variance inflation factor (VIF) was not larger than 4.425 for all variables (using the vif function in the R “car” package [61]) which indicates that none of the predictors have strong linear relationships with the others [62]).

Using a backward model selection, the final model contained the interaction of the text pair and imagery type of pages, the mean log frequency of words, the ratio of verbs and the ratio of adjectives (see Table 4).

**Table 4:** Estimates, T-values and P-values of the Fixed Effects of the Final Model in the Analysis of Reading Speed per Page

Fixed effect	Estimate	*T*	*χ^2^*	*p*
Intercept	2.322	61.295		
Imagery type:	0.030	1.588		
Text-pair: Davis	0.008	0.151		
Mean word frequency	0.024	3.502	10.64^#^	0.001^#^
Ratio of verbs	0.019	3.345	10.373^#^	0.001^#^
Ratio of adjectives	-0.022	-3.697	11.988	<0.001
Imagery type x Text-pair	-0.066	-3.028	8.071	0.005

*Note*. In the two last columns, χ^2^ and p-values were calculated by model comparison. The ^#^ shows parameters which were evaluated by comparing models in which the random slope was removed because the models failed to converge with random slope included.

The full (starting model) was

$$\begin{array}{l} \text{readingspeed \textasciitilde{}imagerystyle*text-pair+}\\ \text{mean\_imageability+mean\_aesthetic\_potential+}\\ \text{mean\_arousal+mean\_logfrequency+mean\_ari+}\\ \text{ratio\_verbs+ratio\_adjectives+}\\ \text{number\_of\_words\_on\_page+page\_number+}\\ \text{(1+imagery\_type|Participant)+(1|item),} \end{array}$$

the final model was

$$\begin{array}{l} \text{readingspeed \textasciitilde{} imagery- style*text-pair+}\\ \text{mean\_logfrequency+ ratio\_verbs+}\\ \text{ratio\_adjectives+ }\\ \text{(1+imagery\_type|Participant)+(1|item).} \end{array}$$

Significance of the predictor variables was evaluated again by model-comparison in which the final model was compared to a similar model without the predictor variable.

**Figure 3:**
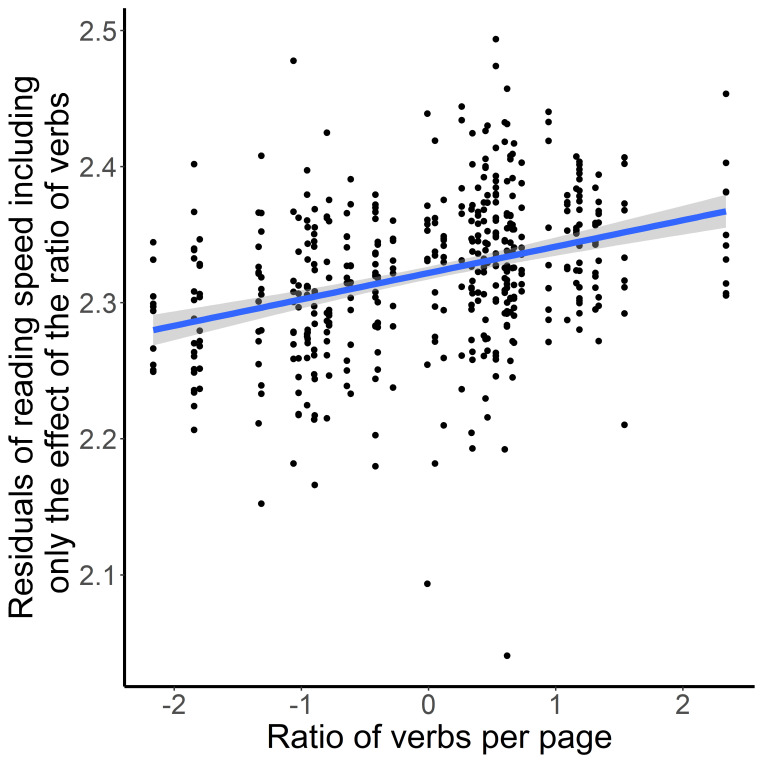
Ratio of Verbs per Pages (X-axis) and Residuals of Reading Speed (Y-axis)

*Note*. The y-axis shows the residuals of the final model when the effect of all variables are removed except the variable of interest (in this case: ratio of verbs per page) [63]).

**Figure 4:**
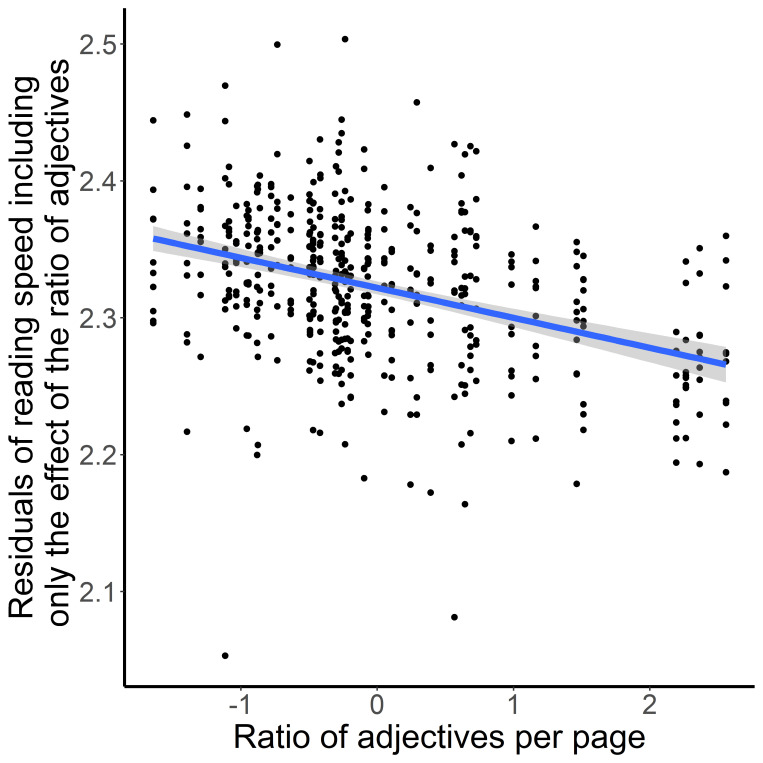
Ratio of Adjectives per Pages (X-axis) and Residuals of Reading Speed (Y-axis)

*Note*. For more explanation, see note of Figure 3.

In the final model, the average log frequency of words affected the reading speed of the pages. Pages with higher mean word frequency were read faster. Pages which contained more verbs were also read faster (Figure 3), while pages with more adjectives were read slower (Figure 4).

**Figure 5:**
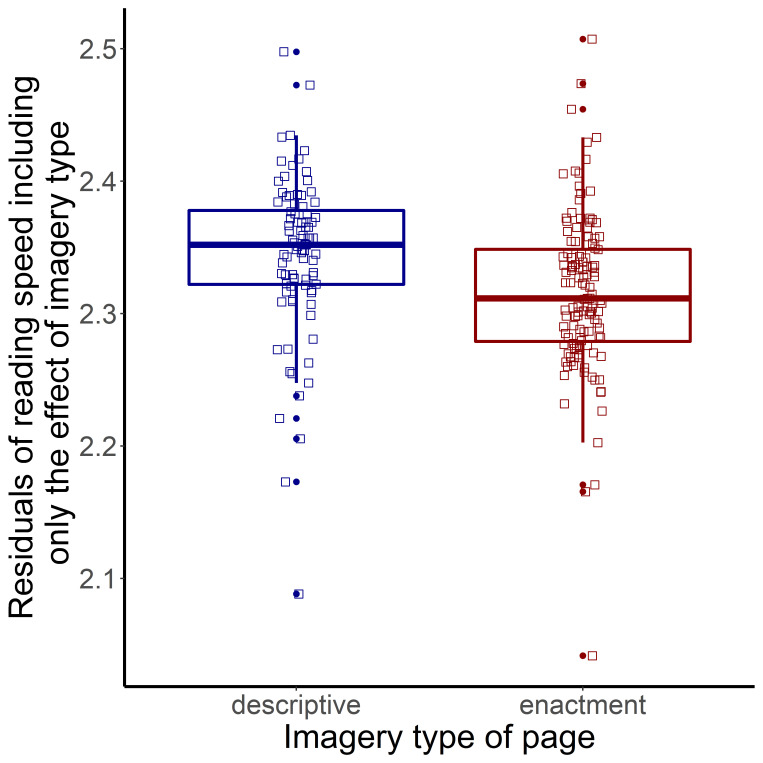
Imagery Type of Pages (X-axis) and Residuals of Reading Speed (Y-axis) in the Davis Texts

*Note*. For more explanation, see caption of Figure 3.

For examining the interaction effect between text pair and imagery type of pages, two mixed-effect models were created separately for the two text pairs (French texts and Davis texts). The predictor variables of the final model (Table 4) were included as fixed effects (except for the variable: text pair). Participants and pages were included as random effects. Imagery type was not included as random slope because the models did not converge. A second model contained the same predictor variables except for imagery type. These two models were compared to evaluate the effect of imagery type in both text pairs, separately. Predictor variables were standardized separately for each dataset. Model comparison showed no effect of imagery type of pages for the French texts (Estimate=0.014, *t*=0.718, *χ^2^*=0.510, *p*=0.475), while it showed a trend for the Davis texts (Estimate=-0.029, *t*=-2.046, *χ^2^*=3.800, *p*=0.051). The pages of the Davis text pair with enactive style were read more slowly (Figure 5).

Analysis of mean fixation duration of words, first fixation time, gaze duration and dwell time.

We included altogether 395 words of the Davis text-pair for the analysis of the mean fixation duration (*mean*=233.795 ms, *sd*=86.280). 96% of the analysed words had a fixation by more than 3 participants. 41% of the analysed fixation duration fall on nouns, 26% on adjectives and 32% on verbs. 42% of the fixations fall on words which part of a descriptive text. We examined the mean fixation duration of these words. Outlier values (values smaller or larger than two times of the standard deviation from mean) were rejected by participants. The predictor variables were 1) word length (based on the number of letters of the words), 2) the automated readability index (ARI) of the sentence which contained the word, 3) the arousal value of words, 4) the aesthetic potential value of words, 5) the imageability value of words, 6) the log-transformed value of word frequency, and categorical variables as 10) word category (whether it was a noun, verb or adjective) and 11) imagery type of the word (whether it was distinctive of the enactive or descriptive style). More explanation of some of the variables is included in the Reading speed of pages section. We also included interaction between word-category and imagery type of words in the starting model. All predictor variables were standardized. Although there was a high correlation between logfrequency and arousal values of words (*r*=-0.834, *p*<0.001), we did not exclude any of these variables as they were not the focus of our analysis. VIF was not larger than 6.466 for all variables.

The final model contained the log frequency of words, the word category and imagery type of words (see Table 5).

**Table 5:** Estimates, T-values and P-values of the Fixed Effects of the Final Model in the Analysis of Mean Fixation Duration per Word

Fixed effect	Estimate	*t*	*χ^2^*	*p*
Intercept	253.433	35.596		
Imagery type: Descriptive	-26.103	-5.609	16.552	<.001
Word category: verbs vs adjectives	-12.110	-2.324	14.529	.002
Word category: verbs vs nouns	-15.823	-3.441		
Log frequency	-9.821	1.981	23.794	<.001

*Note*. In the two last colums, *χ^2^* and *p*-values were calculated by model comparison.

The full (starting model) was

$$\begin{array}{l} \text{mean\_fixation\_duration \textasciitilde{} }\\ \text{imagery type*word-category+}\\ \text{imageability+logfrequency+ aesthetic\_potential+}\\ \text{arousal+word-length+ ari+ }\\ \text{(1+imagery\_type|Participant)+(1|Word),} \end{array}$$

the final model was

$$\begin{array}{l} \text{mean\_fixation\_duration \textasciitilde{} }\\ \text{imagery type+word-category+ logfrequency+}\\ \text{(1+imagery\_type|Participant)+(1|Word).} \end{array}$$

**Figure 6:**
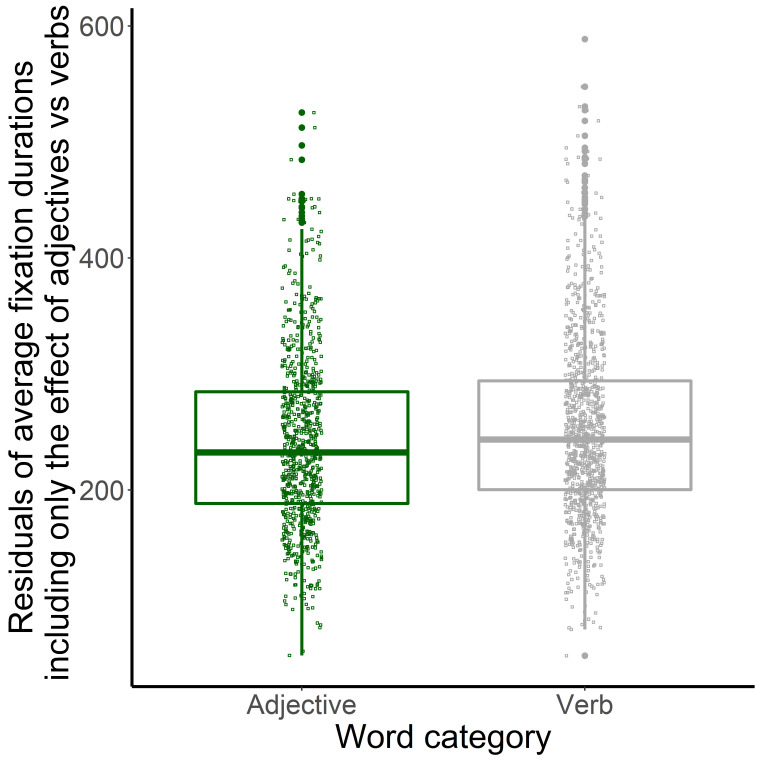
Word-category (Adjectives and Verbs) (X-axis) and Residuals of Mean Fixation Duration (Y-axis) in the Davis Texts

*Note*. For more explanation, see caption of Figure 3.

**Figure 7:**
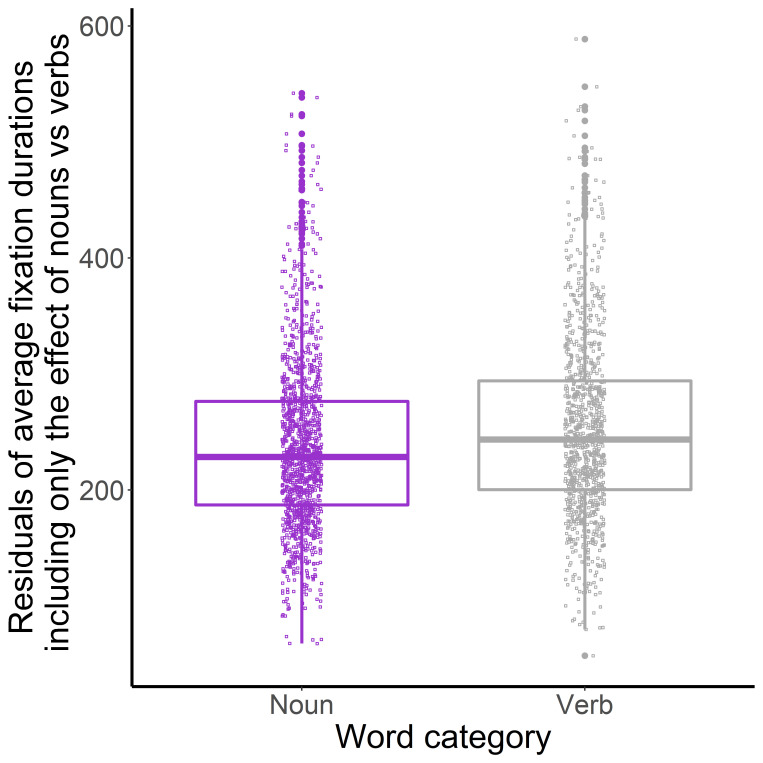
Word-category (Nouns and Verbs) (X-axis) and Residuals of Mean Fixation Duration (Y-axis) in the Davis Texts

*Note*. For more explanation, see caption of Figure 3.

**Figure 8:**
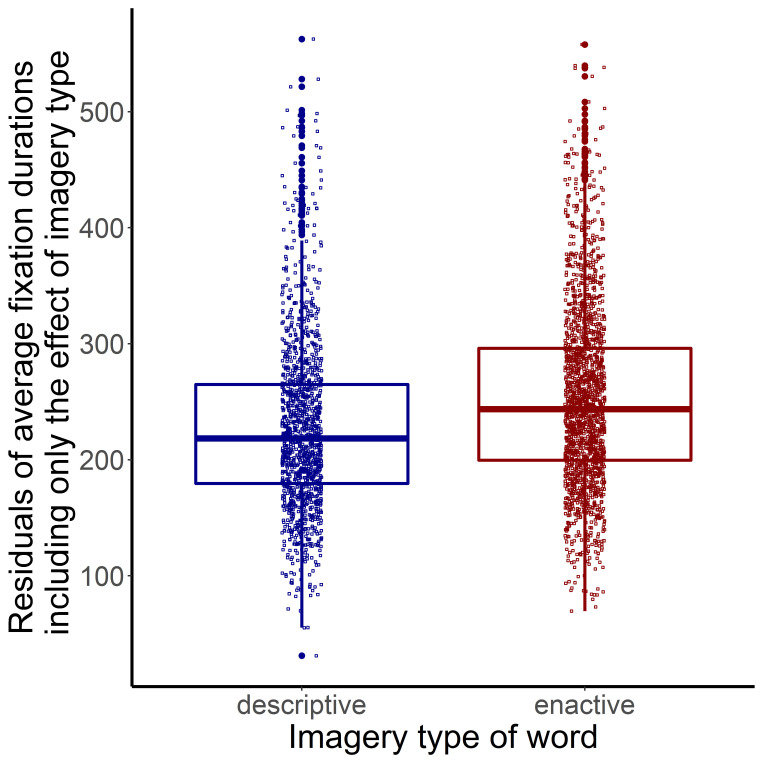
Imagery Type of Words (X-axis) and Residuals of Mean Fixation Duration (Y-axis) in the Davis Texts

*Note*. For more explanation, see caption of Figure 3.

Words with higher frequency had a shorter average fixation duration. To examine the effect of word category, we ran mixed-effect models separately for the pairwise comparison of each word-category. A model where the predictor variables of the final model (Table 5) were included as fixed effects, but one of the word categories was not included in the dataset. Participants and pages were included as random effects. A second model contained the same predictor variables except for word-category. These two models were compared to evaluate the effect of two word categories, respectively. Adjectives and nouns also had shorter fixations than verbs (adjectives vs verbs: *β*=10.776, *t*=2.136, *χ^2^*=4.536, *p*=.033, see Figure 6; nouns vs verbs: *β*=16.950, *t*=3.642, *χ^2^*=12.926, *p*<.001, see Figure 7). There was no difference in the effect of nouns and adjectives on mean fixation duration (*β*=-3.886, *t*=-0.781, *χ^2^*=0.608, *p*=.436). Words which were indicative of description-imagery also had shorter fixations compared to enactive words (Figure 8). Correlation of Imagery type as a fixed effect was smaller than 0.07 with all other fixed effect variables.

In order to provide more detailed measures, we also analysed first fixation duration, gaze duration and dwell time for the two Davis texts. Similarly to the earlier analysis, nouns, adjective and verbs were included. Model reduction followed the same steps as earlier. For each analysis, outliers were rejected per participant. For all analyses, we used the same predictors as earlier and all predictors were standardized. VIF values were not larger than 6.863 for the analysis of first fixation duration, not larger than 7.698 for gaze duration and not larger than 5.301 for dwell time. Predictors of the final models of the analyses of first fixation duration, gaze duration and dwell time are shown in Table 6.

**Table 6:** Estimates, T-values and P-values of the Fixed Effects of the Final Model in the Analysis of First Fixation Duration, Gaze Duration and Dwell Time per Word

Fixed effect	Estimate	*t*	*χ^2^*	*p*
First fixation duration				
Intercept	253.097	33.096		
Imagery type: Descriptive	-24.553	-5.269	33.844^#^	<..001^#^
Word-category: verbs vs adjectives	-9.915	-1.804	11.597^#^	.003^#^
Word-category: verbs vs nouns	-16.618	-3.425		
Log frequency	-9.679	-4.628	20.775	<.001
Gaze duration				
Intercept	303.958	23.680		
Imagery type: Descriptive	-31.422	-3.875	14.723	<.001
Log frequency	-22.194	-3.968	15.368	<.001
Word length	27.300	4.916	23.417	<.001
Dwell time				
Intercept	357.459	17.121		
Imagery type: Descriptive	-24.540	-1.886	7.1357^#^	.008^#^
Log frequency	-23.451	-3.851	14.534	<.001
Word length	43.653	7.137	47.559	<.001

*Note*. In the two last columns, *χ^2^* and *p*-values were calculated by model comparison. The ^#^ shows parameters which were evaluated by comparing models in which the random slope was removed because the models failed to converge with the random slope included.

To sum up, all analyses related to measures of early and late processes on the word-level showed an effect of imagery type. When the words were part of a descriptive passage, mean fixation duration, first fixation duration, gaze duration and dwell time got shorter.

##### Analysis of revisits at word-level

Revisits of words were analysed only for words from the two Davis texts satisfying the criteria mentioned in the method section. We analysed the same words which were also included in the analysis of average fixation duration. Altogether 24,7% of the analysed words had a revisit. We examined all predictor variables by including them in the model in a step-wise manner in the following order: word-length, ARI, arousal, aesthetic potential, imageabilty, logfrequency, word-category and imagery style. Only variables with a significant effect remained in the model

**Figure 9:**
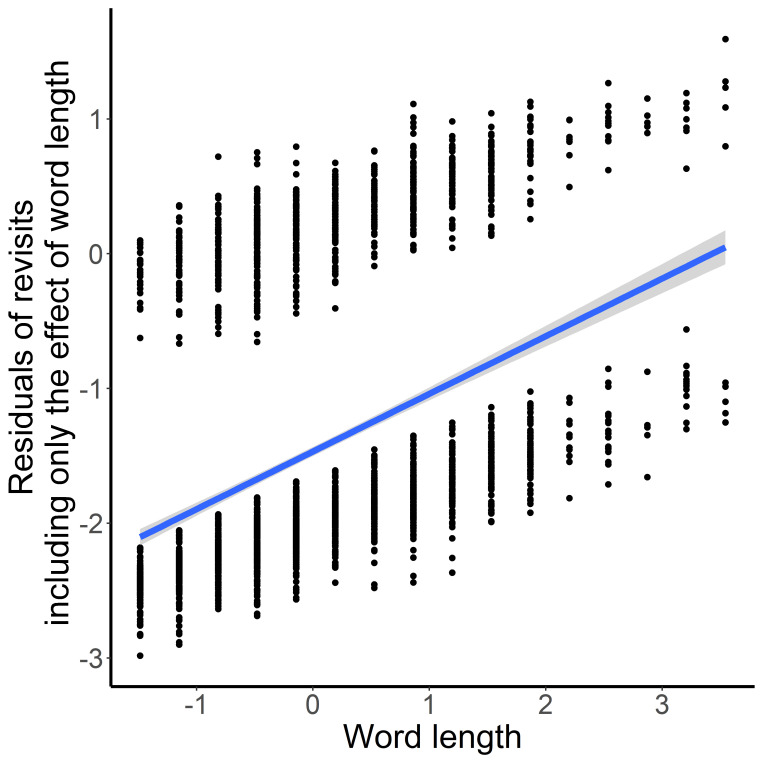
Length of Words (X-axis) and Residuals of Revisits (Y-axis) in the Davis Texts

*Note*. For more explanation, see caption of Figure 3.

The final model only contained word length (Estimate=0.399, *z*=7.522, *p*<.001) indicating more revisits for longer compared to shorter words (Figure 9). In contrast to the duration-based measures no significant effect for imagery style was observed.

## Discussion

In this study, we examined texts with enactive and descriptive imagery cues as described by Kuzmičová [23]. Kuzmičová assumed that during enactment-imagery the reader instantaneously adopts the inner perspective of a story character, prototypically but not necessarily through textual references to object-directed bodily movement. During description-imagery, readers visually imagine a situation as if from the outside, prototypically by following a narrator’s more or less elaborate instructions to visualize a given object or scene. For our study, we selected texts with dominantly descriptive and enactive style, and also indicated for each page and for parts of texts (word, phrases, sentences) which type of imagery they prompted. Four longer excerpts of novels were chosen, one pair of texts each authored by a different French author (here, called French texts), and one pair of texts authored by the same American author (Davis texts). The German translations of these two pairs of texts (each containing an Enactive and a Descriptive Text) were read by different participants.

We expected that Enactive Texts would contain more verbs (as many verbs refer to movements) while there would be more nouns and adjectives in Descriptive Texts. Our analysis of categories of words showed that there were indeed more verbs in both Enactive Texts compared to the Descriptive Texts. The Enactive Text in the French text-pair also contained fewer nouns and adjectives than its descriptive counterpart. When only those selected parts of all texts (words, phrases or sentences) were examined which were expected to distinctly prompt the two different types of imagery, we only found significant differences between word categories within the French but not within the Davis texts. Although the differences between word categories occurred in the predicted direction, it also seems that the relative ratio of the three word categories, verbs, nouns and adjectives, was not a particularly strong textual cue for these imagery styles, as there were smaller differences between them in the texts which were written by the same author (Davis texts). Finer distinctions between categories of words, for example, distinctions between semantic categories of verbs (e.g., verbs referring to movements or mental events; verbs with or without human agent) and nouns (e.g., animate or inanimate nouns) might be more suitable candidates for textual cue analysis in a future study. Such distinctions require a qualitative semantic analysis while we used only automatic procedures to determine syntactic categories of words in our study.

We also tested whether Kuzmičová’s [1, 23] typology was reflected in the self-reports of readers and in the processing of the text. We hypothesized that narrative texts with features prompting dominantly enactment-imagery would lead to higher immersion and transportation during reading and therefore would be accompanied by shorter fixations and faster reading, as also predicted by the NCPM [46, 47]. Texts with descriptive style, however, were expected to elicit a less fluent reading mode with longer fixations. First, it is important to note that participants had to answer comprehension questions after each text and we found no differences in their performance between Enactive and Descriptive Text in any of the two text pairs. Therefore, any differences in participants’ reading experience were probably not caused by difficulties with comprehension of the texts.

Regarding participants’ self-reported reading experience, we found that one item of our reading experience questionnaire showed a difference between Enactive and Descriptive Texts for both text pairs. Participants found it more difficult to imagine the story visually when they read the Descriptive Texts. Hence, Descriptive Texts might have required more conscious effort from the readers’ imagery process, as in following a narrator’s instructions to visually imagine a situation from the outside, while it was easier to adopt the inner stance of characters as invited by the Enactive Texts, an experience likely mediated by processes such as sensorimotor simulation. In light of the fact that both texts in the Davis pair were told from a first-person perspective, this difference in readers’ felt effort to visually imagine cannot – pace Hartung et al. [29] – be related to narrative perspective *per se*. The above interpretation is further supported by another item of the reading experience questionnaire in which readers found that it was more true for the Enactive Texts that sensory images came to them “automatically” during reading. (Although the difference between Enactive and Descriptive Texts was significant in the French text pair, it only reached a trend in significance for this item in the case of the two Davis texts.)

We also predicted that Enactive Texts would elicit higher immersion and transportation, however, items concerning these experiences did not show any difference between text types in either of the two text pairs. Hence, it seems that the relative ease of the imagery process while reading the Enactive Texts did not automatically allow for the readers to experience higher immersion and transportation. This result suggests that felt experiences of imagery on the one hand and immersion and transportation on the other, especially when measured with self-reports after the entire reading act, can be independent from each other, despite commonly found associations [4, 5].

The reading experience questionnaire also showed that the two French texts seemed to differ in many aspects of experience (e.g. suspense, cognitive involvement) and not only in imageability. This was likely due to pronounced differences between the two authors’ styles of writing. Therefore, the two Davis texts written by the same author proved to be a better text material for testing for differences of enactive and descriptive style effects in eye-movements, as these texts differed only in the ease of imagery in the readers’ subjective experiences.

Regarding eye-movements, we predicted that Descriptive Texts would elicit a less fluent reading mode, with slower reading and longer fixations – indicative of the “aesthetic” route in the NCPM [46, 47]. However, we found that reading speed of pages was mainly influenced by word frequency and categories of words, showing a trend for a difference between enactive and descriptive pages only among the Davis texts. Moreover, the enactive pages of the Davis texts were read slower than the descriptive pages. In the Introduction we pointed out that Description-imagery might partly overlap with perceptual simulation examined in other studies. Therefore, this result is perhaps somewhat surprising, given that Mak and Willems’ [16] found that passages cueing perceptual simulation were read slower than passages cueing motor simulation. Moreover, readers did not in fact read the descriptive pages more slowly in spite of the reader’s difficulty of imagery as experienced in the Descriptive Texts in our study. Another difference from Mak and Willems [16] was that we also included word category as a predictor in our analyses, thus allowing to test the effects of imagery style and word category at the same time. As reported, the ratio of adjectives as well as the ratio verbs are significant predictors. Pages with fewer adjectives were read faster compared to pages with more adjectives whereas pages with more verbs were read faster than pages with fewer verbs. These data show that readers are sensitive to different text information. In general, that nicely fits with the assumption of Kuzmičová [1], that mental imagery during reading is prompted by specific features of the text like the usage of action verbs or adjectives. However, how the usage of different word categories influenced mental imagery and how that influences the degree of immersion could not be answered by this study as we observed consistent differences between enactive and descriptive text passages, neither in the analysis of text features nor in the analysis for self-reported immersion. Only for the ease of conjuring mental images, our participants reported consistent higher ratings for enactive compared to descriptive texts. To disentangle the relationship between word category, imagery and immersion, future studies designed to test the predictions of Kuzmičová’s theory as well as the predictions of the NCPM will have to make sure that one type of text indeed causes higher degrees of immersion while the other clearly possesses more aesthetic stylistic features like metaphor, personification or oxymora. This requires the combination of tools for both qualitative and quantitative text analysis, which are still in development for complex literary texts [60].

In order to test whether the observed differences in reading speed per page were caused by longer or shorter fixation duration and / or by more revisits, we also analysed eye-movement behaviour on the word-level for the Davis texts. We found that words which were part of enactive passages were read with longer average fixation duration. More fine grained analysis indicated that this increase occurred in measures reflecting early processes related to visual word recognition and orthographic/lexical access, i.e., in first fixation duration and gaze duration, as well as in measures capturing both early processes and later processes associated with comprehension difficulties, i.e., in dwell time. Comparing the esimates of imagery type observed for gaze duration and dwell time indicated a slightly smaller effect for dwell time compared to gaze duration. Together with the fact that no effect of imagery type could be observed for the probability of revisits, we conclude that imagery type has a stronger influence on early compared to late processes of comprehension. This pattern is in line with the self reported rating data, where no significant effect was observed for the two Davis texts in the scale Ease of Cognitive Access. We therefore assume that the longer fixation duration especially in first fixation and gaze duration and the therefore decreased reading speed observed for enactive passages are linked to the reported differences in mental imagery. For the two Davis texts our participants reported that it was easier to create visual imageries for the enactive compared to descriptive text. Usually, easy to perform processes are associated with faster processing. So why did we observe longer fixations for the enactive compared to the descriptive passages in the two Davis texts? Kuzmičová’s model [1] defined mental imagery as sensorimotor simulation that has become temporarily *conscious*. Accordingly, mental imagery is a controlled and therefore time- and resource-consuming process. We interpreted the longer fixations observed for enactive texts as an indicator for more imagery processing whereas the difficulty in conjuring imagery experienced with the Descriptive Texts could cause lower involvement of imagery processes overall. Based on our fine-grained analysis at word-level we assume that mental imagery defined as a time- and resource-consuming process interferes especially with early and more automatic processes of reading whereas later processes usually associated with comprehension difficulties are less affected.

In this study, we tried to bridge the gap between literary theory and experimental research. Therefore, our starting point was Kuzmičová’s [1] phenomenological typology of narrative styles. We showed that tendencies in texts towards enactive vs. descriptive style [1, 23] influence reader’s experiences of imagery. Descriptive Texts are experienced as more difficult to imagine, while Enactive Texts, which invite readers to experience the inner stance of characters in a multisensory manner through references to object-directed movement, more strongly involve mental imaging. We also found evidence that greater experienced ease of imagery and perhaps more involvement in imaging correlates with slower reading speed and longer average fixation duration, first fixation duration, dwell time and gaze duration. Using the enactive and descriptive categories proved to be a fruitful approach to the study of imagery. It would be also interesting to explore other narrative styles of Kuzmičová’s [1] typology in future experimental research.

Overall, our findings suggest that mental imagery prompted by Enactive Texts such as our stimuli, which were devoid of suspenseful plot or other overt qualities of a page-turner [11, 64], can become a phenomenon distinct from highly immersive or “superficial” modes of perusal. In other words, adopting the inner sensory stance of a character poses no small cognitive task in itself, also on the level of conscious experience. The skill to wilfully tackle this task, and thus become a more proficient reader in the long run [3], can at least partly be honed through practice [2]. As confirmed by our study, however, texts steeped in very detailed descriptions of the sensory world (Descriptive Texts) seem less suited for exercising mental imagery than texts that portray humans interacting freely with its furnishings (Enactive Texts).

## Limitations and Outlook

Our study focused on imagery processes during reading. In order to control for author’s writing style, we used one pair of two excerpts from novels written by two different authors and one pair of excerpts written by the same author. The texts written by the same author provided better examples for testing predictions between the two studied style/imagery types. However, the findings require replication and further research with a more diverse set of texts.

## Ethics and Conflict of Interest

We declare that the contents of the article are in agreement with the ethics described in http://biblio.unibe.ch/portale/elibrary/BOP/jemr/ethics.html and that there is no conflict of interest regarding the publication of this paper.

## Appendix

### Stimuli

#### Practice text (excerpt written by Thomas Mann)

##### Auszug aus Buddenbrooks

Therese Weichbrodt war bucklig, sie war so bucklig, daß sie nicht viel höher war als ein Tisch. Sie war 41 Jahre alt, aber da sie niemals Gewicht auf äußere Wohlgefälligkeit gelegt hatte, so ging sie gekleidet wie eine Dame von 60 bis 70 Jahren. Auf ihren grauen, gepolsterten Ohrlocken saß eine Haube mit grünen Bändern, die über die schmalen Kinderschultern hinabfielen, und nie war an ihrem kümmerlichen schwarzen Kleidchen etwas wie Putz gesehen worden … ausgenommen die große, ovale Brosche, auf der in Porzellanmalerei das Bild ihrer Mutter prangte.

Das kleine Fräulein Weichbrodt besaß kluge und scharfe braune Augen, eine leichtgebogene Nase und schmale Lippen, die sie aufs entschiedenste zusammenpressen konnte … Überhaupt lag in ihrer geringen Figur und allen ihren Bewegungen ein Nachdruck, der zwar possierlich, aber durchaus respektgebietend wirkte. Dazu trug in hohem Grade auch ihre Sprache bei. Sie sprach mit lebhafter und stoßweiser Bewegung des Unterkiefers und einem schnellen, eindringlichen Kopfschütteln, exakt und dialektfrei, klar, bestimmt und mit sorgfältiger Betonung jedes Konsonanten. Den Klang der Vokale aber übertrieb sie sogar in einer Weise, daß sie z. B. nicht »Butterkruke«, sondern »Botter«- oder gar »Batterkruke« sprach und ihr eigensinnig kläffendes Hündchen nicht »Bobby«, sondern »Babby« rief. Wenn sie zu einer Schülerin sagte: »Kind, sei nich–t sa domm!« und zweimal dabei ganz kurz mit dem gekrümmten Zeigefinger auf den Tisch pochte, so machte dies Eindruck, das ist sicher; und wenn Mademoiselle Popinet, die Französin, sich beim Kaffee mit allzuviel Zucker bediente, so hatte Fräulein Weichbrodt eine Art, die Zimmerdecke zu betrachten, mit einer Hand auf dem Tischtuch Klavier zu spielen und zu sagen: »Ich wörde die ganze Zockerböchse nehmen!« daß Mademoiselle Popinet heftig errötete …

Als Kind – mein Gott, wie winzig mußte sie als Kind gewesen sein! – hatte Therese Weichbrodt sich selber »Sesemi« genannt, und diese Änderung ihres Vornamens hatte sie beibehalten, indem sie den besseren und tüchtigeren Schülerinnen, Internen sowohl wie Externen, gestattete, sie so zu nennen. »Nenne mich ›Sesemi‹, Kind«, sagte sie gleich am ersten Tage zu Tony Buddenbrook, indem sie sie kurz und mit einem leicht knallenden Geräusch auf die Stirn küßte … »Ich höre es gern.«

#### Experimental text: Enactive (original excerpt written by Lydia Davis, slightly modified)

Ich machte mit einigen Freunden eine Wüstentour, nichtweit von der Stadt, wo er gelebt hatte, und ich beschloss, bei seiner letzten Wohnadresse vorbeizuschauen.

Ich fuhr alleine los und kaufte mir eine Straßenkarte. Ich studierte sie auf einer Steinmauer, die sich unter mir kalt anfühlte, obwohl die Sonne warm war. Ein Fremder erklärte mir, die von mir gesuchte Straße sei zu weit weg um den Weg zu Fuß zu schaffen, aber ich ging dennoch. Jedes Mal, wenn ich dann auf einer Hügelkuppe stand, blickte ich hinaus aufs Wasser und sah Brücken und Segelboote. Jedes Mal, wenn ich in ein weiteres kleines Tal hinabstieg, schlossen sich die weißen Häuser wieder um mich.

Ich hatte nicht gewusst, wie weitläufig mir die Stadt beim Gehen vorkommen oder wie sehr meine Beine ermüden würden. Ich hatte nicht gewusst, wie sehr mich der Widerschein der Sonne auf den weißen Häuserfronten nach einer gewissen Zeit blenden würde, wie sie Stunde um Stunde auf Häuserfronten hinknallen würde, die zuerst immer weißer wurden und dann, während die Stunden vergingen und meine Augen zu schmerzen begannen, immer weniger weiß. Ich nahm einen Bus und fuhr ein Stück, stieg aus und ging wieder zu Fuß weiter. Obwohl die Sonne den ganzen Tag geschienen hatte, waren die Schatten später am Nachmittag kühl. Ich ging an ein paar Hotels vorbei. Ich wusste nicht genau, wo ich war, aber dann, als das Viertel hinter mir lag, sah ich, wo ich gewesen war.

Nachdem ich ein paar Mal die richtige Richtung eingeschlagen hatte und dann wieder die falsche, landete ich schließlich in seiner Straße. Es war die abendliche Rushhour. Männer und Frauen in Arbeitskleidung gingen straßauf, straßab an mir vorüber. Der Verkehr war zähflüssig. Die Sonne stand tief und das Licht auf den Gebäuden war dunkelgelb. Ich war überrascht. Ich hatte mir nicht vorgestellt, dass sein Viertel so aussehen würde. Ich hatte nicht einmal geglaubt, dass es die Adresse überhaupt gab. Aber da stand das Haus, dreigeschossig, hellblau angestrichen, ein bisschen schäbig.

Seit ich diese Anschrift vor nunmehr über einem Jahr in mein Adressbuch eingetragen hatte, hatte ich mir, als hätte ich das geträumt, eine sehr genaue Vorstellung von einer kleinen, sonnigen Straße mit zweigeschossigen gelben Häusern gemacht, in denen Menschen ein und aus gingen, ein paar Treppen zu einem Treppenabsatz hochstiegen und von da herunterkamen, und ich hatte mir vorgestellt, wie ich selbst an der anderen Straßenseite, schräg gegenüber von seinem Haus, in einem Auto säße und seine Eingangstür und seine Fenster beobachten würde. Ich hatte mir vorgestellt, wie er, mit gesenktem Kopf und gedankenverloren, aus dem Haus treten und eilig die Treppe herunterlaufen würde, oder aber wie er, langsamer, neben seiner Frau die Treppe herunterkäme, nachdem ich ihn davor schon zweimal, ohne dass er davon wüsste mit seiner Frau gesehen hatte, einmal aus der Ferne, als sie auf dem Gehsteig vor einem Kino standen, und einmal durch den Regen hinter seinem Apartmentfenster.

Ich war mir nicht sicher, ob ich mit ihm reden wollte, denn wenn ich mir das vorstellte, dann machte mich der Ärger, den ich dabei auf seinem Gesicht bemerkte, unruhig. Überraschung, dann Ärger und dann Furcht, weil er Angst vor mir hatte. Sein Gesicht war leer und starr, er hatte seine Lider gesenkt und seinen Kopf ein wenig in den Nacken zurückgeworfen: Was wolle ich ihm denn nun schon wieder antun? Und er würde einen Schritt zurück tun, als wäre er damit tatsächlich schon vor mir sicher.

Obwohl ich sah, dass es sein Haus wirklich gab, glaubte ich nicht, dass es sein Apartment geben würde. Und auch wenn es sein Apartment gab, so glaubte ich nicht, dass sich auf einem Klebeband neben der Klingel sein Name finden würde. Ich überquerte die Straße und betrat das Haus, in dem er vielleicht noch bis vor kurzem gelebt hatte, sicherlich aber irgendwann während des letzten Jahres, und las die Namen ARD und PRUETT auf einem weißen Kärtchen neben der Klingel für die Nummer 6, sein Apartment.

Später wurde mir klar, dass es dieses sonderbare geschlechtslose Paar, Ard und Pruett, gewesen sein musste, das all das auffand, was er zurück gelassen hatte: die Reste von Klebeband, die an Dingen hafteten, die Büroklammern und Reißzwecken Zwischen den Dielenbrettern, die Topflappen oder Gewürzfläschchen oder Kochtopfdeckel hinter dem Herd, den Staub und die Brotkrümel in den Schubladenecken, die harten, verschmutzten Reinigungsschwämme unter der Badewanne und der Küchenspüle, mit denen er in seiner zupackenden Art ein Waschbecken oder die Küchentheke gesäubert hatte, die herrenlos in den dunklen Regionen des Wandschranks hängenden Kleidungsstücke, Holzsplitter, Löcher im Putz, die von Nägeln herrührten, und deren verschmierte und zerkratzte Umgebung, etwas, das Ard und Pruett schon allein deshalb willkürlich vorgekommen sein musste, weil sie nicht wissen konnten, wozu es da gewesen war. Obwohl sie mich nicht kannten und ich ihnen noch nie begegnet war, spürte ich plötzlich, dass zwischen mir und diesen beiden Menschen eine Verbindung bestand, denn auch sie hatten in einer intimen Beziehung zu ihm gelebt. Natürlich konnten es auch ihre Vormieter gewesen sein, die vorgefunden hatten, was er hinterlassen hatte, und vielleicht waren Ard und Pruett also auf die Spuren von jemand ganz anderem gestoßen.

Weil ich alles tun musste, um ihn zu finden, drückte ich auf ihren Klingelknopf. Fand ich ihn diesmal nicht, würde ich es bleiben lassen. Ich klingelte wieder, und wieder, und dann noch einmal, aber nichts rührte sich.

Ich stand draußen auf der Straße gerade so lange, bis sich das Gefühl eingestellt hatte, ich sei endlich angekommen - am Ende einer unvermeidlichen Reise.

Ich war zu Fuß zu einem Ort aufgebrochen, der für einen Fußmarsch zu weit entfernt war. Ich war immer weiter gegangen, obwohl es schon zu spät und ich an der Grenze meiner Kraft war. Ein Teil meiner Kräfte kehrte zurück, als ich zu dem Ort kam, wo er gelebt hatte. Nun ging ich an seinem Haus vorbei, weiter in Richtung Chinatown und zum Rotlichtviertel, in Richtung Lagerhäuser unten an der Bay - und wie ich, bei meinen Anstrengungen, mir das Stadtbild in Erinnerung zu rufen, glaubte in Richtung Wasser, und obwohl er nicht mehr in diesem Haus lebte und obwohl ich so müde war und immer weiter gehen musste und zu allen Seiten immer neue Hügel zu besteigen waren, fühlte ich mich ruhiger werden, weil ich da gewesen war, ruhig, wie ich es, seit er mich verlassen hatte nicht mehr gewesen war - so als hätte ich ihn, obwohl er nicht da war, wieder gefunden.

Vielleicht machte der Umstand, dass er nicht da war, diese Rückkehr möglich und machte ein Ende möglich. Denn wäre er da gewesen, so hätte alles weitergehen müssen. Ich hätte etwas tun müssen, und sei's auch nur weggehen und aus großer Distanz darüber nachdenken. Nun konnte ich meine Suche einstellen.

Aber der Augenblick, in dem mir bewusst wurde, dass ich aufgegeben hatte, als mir bewusst wurde, dass ich meine Suche beendet hatte, kam erst etwas später, während ich in einer Buchhandlung der Stadt saß, im Mund den Geschmack von billigem, bitterem Tee, den mir ein Fremder gebracht hatte. Ich war hierher gekommen, um mich auszuruhen - in einem alten Haus mit knarrenden Fußböden, einer schmalen Treppe, die hinunter führte, einer trüben Kellerbeleuchtung und einem helleren und saubereren Licht im Erdgeschoss. Ich war in der Buchhandlung herumgegangen, war die Treppe hinunter und wieder hochgestiegen und um jeden Bücherschrank gebogen. Ich setzte mich hin, um in einem Buch zu blättern, war aber zu müde und durstig, um zu lesen.

#### Experimental text: Descriptive (original excerpt written by Lydia Davis, slightly modified)

Ich kann mich nicht an den Anlass für den Empfang erinnern. In einer Art sonnendurchflutetem, kreisrundem oder achteckigem Atrium mit davon abzweigenden Eingängen holte mich Mitchell, der mir seinen Namen nannte, aus einer Gruppe von Leuten heraus und brachte mich zu ihm. Ich vergaß den Namen sofort wieder, wie fast immer, wenn ich jemandem vorgestellt wurde. Er wusste schon, wer ich war, also vergaß er meinen Namen nicht. Mitchell ging wieder weg und ließ uns allein. Wir standen da, mitten zwischen Frauen, die langsam und zögernd, einzeln und zu zweit, ins starke Sonnenlicht hinein- und wieder aus ihm heraustraten und in den Räumen umhergingen. Er sagte, er habe sich vorgestellt, ich sei älter. Es überraschte mich, dass er sich überhaupt etwas vorgestellt hatte. Ich war in mehrfacher Hinsicht überrascht: von seiner Freimütigkeit, von der Art seiner Kleidung, die mir wie ein Wander-Outfit vorkam, und mehr noch von der Tatsache, dass es ihn überhaupt gab und er dastand und mit mir redete, wo ihn davor doch noch keiner mir gegenüber erwähnt hatte. Ich dachte nicht mehr an ihn, nachdem ich weggegangen war, vielleicht weil er so jung war.

Später fuhr ich zu einem schäbigen Cafe an der Küstenstraße nördlich der Stadt, in der ich lebte, und er und ein paar Freunde und andere Leute, die ich nicht kannte, waren dorthin gekommen, um irgendeine Aufführung mit primitiven Stammesgesängen zu sehen. Als ich den Raum betrat, war dieser, abgesehen von der Bühnenbeleuchtung, bereits verdunkelt. Der einzige freie Stuhl an dem langen Tisch, den ich entdecken konnte, war der neben ihm, obwohl von der Rückenlehne ein Kleidungsstück und vielleicht eine Handtasche hingen. Als er sah, dass ich wegen des Stuhles zögerte, stand er auf, tat die Sachen weg und trug sie ans andere Ende des Tisches. Bald nachdem die Aufführung in dem trüben Licht begonnen hatte, hielt tatsächlich eine andere Frau auf den Stuhl zu und ging irritiert wieder weg, um sich einen anderen Sitzplatz zu suchen. Ich weiß nicht, wer die Frau war.

Er saß am einen Ende, mit dem Rücken zur Tür, durch die ich gerade herein gekommen war, und sah über den Tisch; ich saß links von ihm, mit Blick auf eine kleine Bühne, auf der zwei Männer eine Vorstellung gaben: einer von ihnen intonierte Stammesweisen und sang, der andere zupfte eine Bassgeige. Mir gegenüber saß Ellie. Ich kannte sie damals nicht sehr gut. Während der Vorstellung, beugte er sich zu ihr hin.

Als wir um halb acht oder acht hinausgingen, war es auf der Straße schon dunkel. Oder, richtiger: die Straßenseite, auf der wir gingen, war von Straßenlampen und den Scheinwerfern rund ums Cafe und die Läden in seiner Nähe beleuchtet, wogegen es auf der anderen Straßenseite dunkel war, weil an ihr entlang die Eukalyptusbäume standen, die das Licht von der Straße fernhielten. Zwischen den Bäumen hingen ein oder zwei Transparente und hinter den Bäumen liefen zwei Paar Eisenbahnschienen nebeneinander her, auch sie dunkel, und quer zu den Schienen verlief ein kleines Bachbett, das selbst nicht sichtbar war, aber durch das hohe Gras, das an seinen Rändern wuchs, seitlich begrenzt wurde, und dahinter, am Fuß eines kahlen Hügels, noch eine Straße, schmäler und nicht stark befahren, aber gut beleuchtet.

Ich glaube, er stolperte, als er in der Dunkelheit die durchfurchte Einfahrt unter der Zeder überquerte, aber da mag ich auch etwas durcheinander bringen. Nun ging ich vorne weg, und an der vorderen Hauswand hob er die herabhängende Ranke einer wild wuchernden Kletterrose hoch, damit ich unterhalb durchgehen konnte, ohne mich zu kratzen. Vielleicht aber hätte er das in der Dunkelheit gar nicht gekonnt, und es passierte an einem anderen Tag, bei Tageslicht. Oder es passierte tatsächlich in dieser Nacht, aber die Nacht war nicht ganz so dunkel. Um die Wahrheit zu sagen: Bloß in meiner Erinnerung ist diese spezielle Nacht so dunkel, denn mir ist bewusst, dass es in der Nähe zwei helle Straßenlampen gab: Eine von ihnen warf ihr Licht direkt in mein Zimmer.

Wir überquerten die kreisförmige Einfahrt und gingen an dem struppigen Rosenstrauch neben dem Fenster vorbei, und vorbei an den Pfennigbäumen rund um das Haus. Wir folgten dann einem mit Backsteinen gepflasterten Weg zu dem weiß angestrichenen Holztor, das in einen weiß angestrichenen Holzzaun eingelassen war, und traten durch das Tor in den Bogengang, der an den Fenstern meines Zimmers vorbei zu meiner Zimmertür führte. Elektrisches Licht fiel aus einer Laterne, die an der Stuckwand neben der Tür hing.

In der Morgendämmerung- ich hatte noch geschlafen- wachte ich auf, weil er irgendetwas zu mir sagte, bevor er ging. Ich wurde noch wacher, um zu verstehen, was er sagte. Er zitierte ein Gedicht - ein Abschiedsgruß, und ich verstand, warum er es tat, aber es ging mir auf die Nerven.

Und wieder heulte sein Wagen auf, als er vor dem Haus losfuhr und den Frieden meiner wohlhabenden Nachbarn störte. Und selbst wenn ihn keiner sah oder hörte, so genierte ich mich dennoch, weil ein so junger Mann im Morgengrauen von meinem Haus wegfuhr, in einem Auto, das mit seinem röhrenden Motor die Stille des eleganten Badeorts störte, als es den Hügel hinunter fuhr, vorbei an den Zäunen und Hecken der Grundstücke meiner Nachbarn: die Leute auf der anderen Straßenseite mit ihrem pagodenartigen Haus, denen ein großes Stück Stadt gehörte und die uns - Madeleine und mich -bei einer anderen Gelegenheit zusammen mit vielen anderen Bürgern der Stadt zu einer Party einluden, weil sie etwas zu feiern hatten: die Einweihung einer Neuanschaffung oder eines Zubaus, vielleicht ihres Swimmingpools; das ältere Paar, das weiter unten am Fuß des Hügels lebte und dessen kunstvoll angelegter Kakteengarten an die schmale Straße grenzte, die zu dem kleinen und feinen Geschäft führte, wo ich so Sachen wie Zigaretten und Katzenfutter kaufte; unsere unmittelbaren Nachbarn, ein junges Paar, das in einem kleinen, weiß gestrichenen Landhaus lebte, das nicht ihr Eigentum, sondern von ihnen gemietet war, was ich damals allerdings nicht wusste- und danach vorbei an der dunkelbraunen norwegischen Holzkirche mit dem Bestand an Eukalyptusbäumen davor, um am Fuße des Hügels rechts abzubiegen.

Ein paar Wochen, bevor ich ihn kennenlernte, war ich in die kleine Stadt übersiedelt. Ich hatte eine Stelle, aber keine Bleibe. Mitchell kannte zwei Adressen, wo es mir seiner Meinung nach vielleicht gefallen würde - ein kleines möbliertes Apartment, das ich für mich alleine hätte, und ein unmöbliertes Zimmer in einem großen Haus, das ich mit einer anderen Frau teilen würde. Er brachte mich zunächst zum Haus - das Apartment sah ich mir danach gar nicht mehr an.

Das Haus war schön und fast leer; es hatte zwei Flügel, deren Zimmer auf eine Terrasse hinausgingen, die von einem Zaun und alten Büschen eingefasst war. Ich dachte, es sehe aus wie eine spanische Hazienda, obwohl ich von einer spanischen Hazienda keine genauen Vorstellungen hatte. Die Frau lebte da mit ihrem Hund und ihrer kleinen Katze. Keiner kannte sie näher, aber alle hatten sich über sie eine Meinung gebildet. Madeleine trat zur Begrüßung aus ihrem Zimmer an der anderen Seite der Terrasse. Sie war groß gewachsen, hatte langes, rötlich-blondes, hinten zusammengebundenes Haar, ein breites, verkrampftes Dauerlächeln auf ihrem Gesicht.

Den Großteil des Tages saß ich an meinem Kartentisch und arbeitete. Mein Zimmer war sehr groß, es hatte rote Bodenfliesen, ein innen offenes Giebeldach mit dunklen Dachbalken, tiefe Fensterlaibungen, weiße Stukkatur an den Wänden, Draußen waren dunkelgrüne Äste einer Kiefer, die sich vor dem Himmel träge hoben und senkten, ein üppiger Strauch mit roten Rosen, jenseits der Bäume, elastische, gezackte Sprosse von Sukkulenten und die weiche puderartige Erde, auf der, im Kreis um die hohe Zypresse, die sich vom Haus wegneigte, verstreut Kiefernzapfen lagen. An der gegenüberliegenden Straßenseite war ein orientalisch verziertes Gittertor.

#### Experimental text: Enactive (original excerpt written by Jean-Phillippe Toussaint, slightly modified)

Es war etwa zur gleichen Zeit in meinem Leben, einem ansonsten ruhigen Leben, als in meinem unmittelbaren Umfeld zwei Ereignisse zusammentrafen, die, jedes für sich, kaum von Belang waren und die, gemeinsam betrachtet, leider nichts miteinander zu tun hatten. Ich hatte gerade beschlossen, den Führerschein zu machen, und mich kaum an diesen Gedanken gewöhnt, als mir mit der Post eine Nachricht zuging: Ein Freund, den ich aus den Augen verloren hatte, teilte mir in einem maschinengeschriebenen Brief, auf einer ziemlich alten Maschine, seine Heirat mit. Wenn ich etwas nicht ausstehen kann, dann sind es die aus den Augen verlorenen Freunde.

Eines Morgens also fand ich mich im Büro einer Fahrschule ein. Es war ein größerer Raum, ziemlich düster, in dessen hinterem Teil, einer Projektionswand gegenüber, mehrere Stuhlreihen aufgestellt waren. An den Wänden hingen alle möglichen Verkehrsschilder und hier und da einige fahlblaue Plakate, verblichen und vom Alter gezeichnet. Die junge Frau, die mich empfing, gab mir eine Liste der Dokumente, die ich für die Anmeldung benötigte, informierte mich über die Kosten, über die Anzahl der Stunden, die ich zu nehmen hätte, etwa zehn für den theoretischen, zwanzig für den praktische Teil, vorausgesetzt, alles lief gut . Dann öffnete sie eine Schublade und hielt mir ein Formular hin, das ich, ohne einen Blick darauf geworfen zu haben, zurückwies, es eile nicht, erklärte ich ihr, lieber würde ich es später ausfülle,wenn möglich zum Beispiel, wenn ich mit den Unterlagen wiederkäme, das erschiene mir viel einfacher.

Den weiteren Tag verbrachte ich bei mir zuhause, las Zeitung, erledigte einige Post. Später am Nachmittag ergab es sich, dass ich zufällig nochmals an der Fahrschule vorbeikam. Ich ergriff die Gelegenheit, öffnete die Tür, und als die junge Frau mich eintreten sah, dachte sie, ich sei zurückgekommen, um nun tatsächlich die Anmeldung vorzunehmen. Ich musste sie enttäuschen, ließ sie aber wissen, dass es mit der Sache voranging, ich hatte schon die Photokopie meines Ausweises und beabsichtigte, in den kommenden Stunden herauszufinden, wie man ein polizeiliches Führungszeugnis bekommt. Ratlos sah sie mich einen Augenblick an und schärfte mir nochmals ein, nicht die Fotos zu vergessen (ja, ja sagte ich , vier Fotos).

Noch am selben Abend, es war mir gelungen das Führungszeugnis zu besorgen (sogar eine Kopie hatte ich mir machen lassen), erschien ich wieder in der Fahrschule. Ich blieb einen Moment an der Tür stehen, den Blick auf die Türglocke gerichtet, ein Glockenspiel aus Kupfer, das ein kleiner Hammer bearbeitete. Lächelnd erklärte mir die junge Frau, daß sie es gewöhnlich abschalte, wenn sie da wäre, und sie erhob sich, umkreiste ihren Schreibtisch, durchquerte in einem hellen und sehr leichten Kleid den Raum und zeigte mir den dazugehörigen Schalter. Ich muß schon sagen, ein ziemlich ausgeklügeltes System, und wir machten uns eine Weile den Spaß, das Läutwerk ab- und anzustellen, die Tür zu öffnen und zu schließen, mal von innen, mal von außen, wo es bereits zu dämmern begann. Wir waren beide gerade draußen, als drinnen das Telefon läutete. Rasch trat sie wieder hinein, und ich wartete, während sie den Anruf beantwortete, ihr gegenüberstehend, schob dabei mit meinen Fingerspitzen Gegenstände über ihren Schreibtisch, öffnete irgendeine Akte. Sie hatte kaum aufgelegt, als sie mich fragte, wie weit ich inzwischen mit der Zusammenstellung meiner Unterlagen sei, und dann sichteten wir gemeinsam die Dokumente, die ich schon zusammengetragen hatte. Außer den frankierten Kuverts fehlten für die Anmeldung offenbar nur noch die Fotos. In diesem Zusammenhang vertraute ich ihr, bevor ich mich verabschiedete übrigens an, dass ich gerade vorher bei mir zu Hause einige Fotos gefunden hätte aus der Zeit, als ich klein war. Ich möchte sie ihnen zeigen , sagte ich, zog einen Umschlag aus meiner Jackentasche und, um ihren Schreibtisch herumgehend, legte ich ihr eins nach dem anderen vor, beugte mich dabei über ihre Schulter und begleitete meine Erläuterungen mit dem Finger. Also hier, sagte ich, stehe ich neben meinem Vater und da, in den Armen meiner Mutter, ist meine Schwester, und hier, das sind wir alle beide, meine Schwester und ich, im Schwimmbad, hinter dem Rettungsring, das ist meine Schwester, ja, ganz klein. Hier, da sind wir wieder, meine Schwester und ich , im Schwimmbad. So, sagte ich und steckte die Fotos wieder in den Umschlag zurück, Sie sind, denke ich, sicher einer Meinung mit mir, daß das für uns nicht von großem Nutzen ist (für die Anmeldung, sagte ich).

Als ich am nächsten Morgen gleich nach Öffnung wieder in meine Fahrschule kam (ich hatte die Fotos immer noch nicht, es war zwecklos, mich darauf anzusprechen), war die junge Frau gerade dabei, auf einer kleinen Wärmeplatte Tee zuzubereiten. Sie trug einen dicken weißen Wollpullover über ihrem Kleid und wirkte völlig verschlafen. Ich setzte mich der Projektionswand gegenüber auf einen der Stühle, schlug eine Zeitung auf und begann zu lesen, um sie nicht zu stören. Während ich mir die Neuigkeiten des Tages zu Gemüte führte, tauschten wir einige Gemeinplätze aus, und als der Tee fertig war, fragte sie mich gähnend, ob ich eine Tasse wolle. Ohne meine Lektüre zu unterbrechen, lehnte ich dankend ab. Gegen ein Tässchen Kaffee allerdings hätte ich nichts einzuwenden, sagte ich und faltete mein Blatt wieder zusammen. Kann auch Nescafe sein, sagte ich. Während die junge Frau Nescafe besorgen ging (holen Sie doch auch gleich ein paar Croissants, sagte ich, wenn Sie schon dabei sind), blieb ich allein in der Fahrschule zurück, und damit ich nicht gestört wurde, hob ich den Riegel an der Glastür und verschloss sie. Ich hatte mich gerade wieder in meine Zeitung vertieft, als ich hinter mir sachte Schläge gegen das Glas hörte. Gequält hob ich den Kopf, drehte mich um und erblickte, nein; nicht etwa die junge Frau, sondern einen jungen Mann, hässlich wie sonst was, mit einer Art grünem Regenmantel und weißen Socken in Slippern. Sorgfältig faltete ich meine Zeitung und stand schließlich auf, um die Tür zu öffnen. Der sollte was erleben, der Knabe. Was wollen Sie, sagte ich. Ich bin gerade achtzehn geworden, sagte er (als ob mich das beeindrucken würde). Es ist geschlossen, sagte ich. Aber ich war gestern schon mal hier, fuhr er fort. Ich wollte nur die Anmeldung abgeben. Jetzt sind Sie mal nicht so bockig, also bitte, sagte ich, sanft die Augenlider senkend. Ich machte wieder die Tür zu. Während er davonschlich, blieb ich für einige Augenblicke hinter der Glastür stehen, die Hände in den Taschen meines Mantels vergraben, und schaute mir versonnen die Aussicht an. Vögel pickten auf dem Gehweg. Etwas weiter weg war der junge Mann inzwischen bei seinem Mofa angekommen und damit beschäftigt, mit einem ausgefransten Gummiriemen seine Unterlagen auf den Gepäckträger zu schnallen. Er drehte sich um, warf nochmals einen Blick in meine Richtung, stieg dann auf sein Mofa und entschwand auf der Straße, wobei er bei der Verfolgung eines Autobusses heftig in die Pedale trat, es war hoffnungslos, echt.

Beim Frühstück, das wir wenig später vor der Projektionswand sitzend einnahmen, wir hatten einen Stuhl vor uns hingestellt und die Tüte mit den Croissants der Länge nach aufgerissen, plauderten die junge Frau und ich über dies und jenes, versuchten, unsere Bekanntschaft zu vertiefen. Mit überkreuzten Beinen saß sie neben mir, die Ärmel ihres dicken Pullovers hochgekrempelt, und massierte sich geistesabwesend einen Arm, den Kopf gesenkt, immer noch verschlafen. Wir sprachen über alles und nichts, in aller Ruhe, tranken gelegentlich einen großen Schluck. Während sie dann aufzuräumen begann, sammelte ich die auf dem Stuhl verstreuten Krümel in meiner hohlen Hand, und als sie mich fragte, was ich heute zu tun gedenke, sagte ich ihr, ich würde vermutlich versuchen, mich um die Fotos zu kümmern. Sie hatte wieder hinter ihrem Schreibtisch Platz genommen, und während sie mit dem Ordnen einiger Papiere beschäftigt war, sagte sie gähnend, bei dem Tempo würde ich es nie schaffen, alle Unterlagen zusammenzutragen. Ich war mir da nicht so sicher. Meiner Meinung nach täuschte sie sich über meine Methode, sie verkannte, dass mein scheinbar recht unerfindliches Spiel der Annäherung, die Wirkung hatte, die Realität, an der ich mich stieß, gewissermaßen zu zermürben, wie man beispielsweise auch eine Olive mürbe machen kann, bevor man sie erfolgreich auf eine Gabel spießt. Und das meine Neigung, nie etwas zu überstürzen, mir durchaus nicht zu meinem Nachteil gereichte, sondern mir in Wirklichkeit eine günstige Ausgangslage verschaffte, von der aus ich, wenn die Dinge mir reif erschienen, zupacken konnte.

#### Experimental text: Descriptive (original excerpt written by Georges Perec, slightly modified)

Zuerst würde der Blick über den grauen Teppichboden eines langen, hohen und schmalen Korridors gleiten. Die Wände wären Einbauschränke aus hellem Holz, deren Messingbeschläge glänzten. Drei Stiche – der eine stellt Thunderbird dar, Sieger in Epsom, der andere einen Schaufelraddampfer, die Ville-de-Montereau, und der dritte eine Lokomotive von Stephenson – würden zu einem von großen, schwarz gemaserten Holzringen gehaltenen Ledervorhang führen, der sich durch eine einfache Handbewegung zurückschieben ließe. Nun würde der Teppichboden einem fast gelben Parkett weichen, das drei Teppiche in gedämpften Farben teilweise bedeckten.

Es wäre ein Wohnraum, etwa sieben Meter lang, drei breit. Links, in einer Art Nische, stünde ein großes schwarzes, abgewetztes Ledersofa zwischen zwei Bücherschränken aus heller Kirsche, in denen die Bände sich kunterbunt übereinanderstapelten. Über dem Sofa nähme eine alte Seekarte die ganze Länge der Wand ein. Hinter einem kleinen niedrigen Tisch, unter einem seidenen, mit drei breitköpfigen Messingnägeln an der Wand befestigten Gebetsteppich, einem Gegenstück zum Ledervorhang, würde ein anderes, mit hellbraunem Samt bezogenes, rechtwinklig zum ersten aufgestelltes Sofa zu einem kleinen hochbeinigen, dunkelrot lackierten Möbelstück mit drei Fächern führen, in dem sich Nippes befände: Achate und Steineier, Schnupftabakdosen, Bonbonnieren, Jadeaschenbecher, eine Perlmuschel, eine silberne Taschenuhr, ein geschliffenes Glas, eine Kristallpyramide, eine Miniatur in ovalem Rahmen. Etwas weiter, nach einer gepolsterten Tür, enthielte ein in die Ecke eingepasstes Wandregal Kästchen und Schallplatten sowie einen geschlossenen Plattenspieler, von dem man nur vier ziselierte Metallknöpfe sähe und über dem ein Stich des Grand Défilé de la fête du Carrousel hinge.

Vom Fenster aus, dessen weiße und braune Vorhänge die Farben des Gemäldes von Jouy aufgreifen würde, entdeckte man ein paar Bäume, einen winzigen Park, ein Stück Straße. Zu einem mit Papieren und Schreibstiften überhäuften Sekretär mit Rollläden würde ein kleiner Rohrsessel gehören. Ein Figurenständer trüge ein Telefon, ein ledernes Notizbuch, einen Abreißblock. Hinter einer anderen Tür und nach einem drehbaren, niedrigen und quadratischen Bücherschrank, auf dem eine große zylindrische, mit gelben Rosen gefüllte blau verzierte Vase thronte, über der wiederum ein länglicher, in einem Mahagonirahmen gefasster Spiegel hinge, würden ein schmaler Tisch und zwei dazugehörige, mit Schottenkaro bezogene Polsterbänke zu dem Ledervorhang zurückführen.

Alles wäre braun, ocker, fahlrot, gelb: eine Welt leicht altmodischer Farben, die Töne sorgfältig, fast pedantisch dosiert, zwischen denen ein paar hellere Flecke, das fast schreiende Orange eines Kissens, ein paar knallbunte zwischen den Ledereinbänden verlorene Bücher überraschen würden. Am hellen Tag würde das in Strömen eindringende Licht diesen Raum trotz der Rosen ein wenig traurig machen. Es wäre ein Raum für den Abend. Im Winter jedoch, bei zugezogenen Vorhängen, einigen Lichtpunkten – die Ecke mit den Bücherregalen, die Schallplattenspieler, der Sekretär, der niedrige Tisch zwischen den beiden Sofas, die undeutlichen Reflexe im Spiegel – und den großen Schattenzonen, in denen alle Dinge aufleuchten würden, das polierte Holz, die schwere, kostbare Seide, das geschliffene Kristall, das weiche Leder, wäre er ein Hafen des Friedens, eine Stätte des Glücks.

Die erste Tür öffnete ein Zimmer, das mit hellem Teppichboden ausgelegt wäre. Ein großes englisches Bett nähme den Hintergrund ein. Rechts, zu beiden Seiten des Fensters, enthielten schmale, hohe Regale Bücher, zu denen man immer wieder greifen würde, Alben, Spielkarten, Keramikschalen, Halsketten, Krimskrams. Links ständen ein alter Eichenschrank und zwei stumme Diener aus Holz und Messing einem kleinen, mit grauer, fein gestreifter Seide bezogenen Sessel und einem Frisiertisch gegenüber. Durch eine halb geöffnete, ins Badezimmer führende Tür sähe man dicke Bademäntel, Messinghähne in Form von Schwanenhälsen, einen großen verstellbaren Spiegel, ein Paar englische Rasiermesser und ihr grünes Lederetui, Flakons, Bürsten mit Horngriffen, Schwämme. Die Wände des Schlafzimmers wären mit bedrucktem Kattun bespannt; über das Bett wäre ein Schottenplaid gebreitet. Ein Nachttisch, an drei Seiten von einem niedrigen Messinggitter eingefasst, trüge einen Silberleuchter mit einem Lampenschirm aus blassgrauer Seide, eine viereckige Stutzuhr, eine Rose in einem Stielglas und, in dem unteren Fach, zusammengefaltete Zeitungen, ein paar Illustrierte. Etwas weiter, am Bettende, läge ein großes Sitzkissen aus Naturleder. An den Fenstern glitten die Tüllgardinen über Messingstangen; die grauen Vorhänge aus dichtem Wollstoff wären halb zugezogen. In der Dämmerung wäre das Zimmer noch hell. An der Wand, über dem schon für die Nacht aufgedeckten Bett, würde zwischen zwei kleinen elsässischen Lampen die verblüffende, schmale und lange Schwarz-Weiß-Fotografie eines am Himmel dahinfliegenden Vogels durch ihre etwas allzu formale Perfektion überraschen.

Die zweite Tür führte in ein Arbeitszimmer. Die Wände wären von oben bis unten mit Büchern und Zeitschriften tapeziert, nur hier und da, um das Einerlei der Buchrücken und Broschüren zu unterbrechen, ein paar Stiche, Zeichnungen, Fotografien – der Heilige Hieronymus von Antonello da Messina, ein Ausschnitt aus dem Triumph des Heiligen Georg, ein Blatt aus den Carceri von Piranesi, ein Porträt von Ingres, eine kleine, mit der Feder gezeichnete Landschaft von Klee, eine vergilbte Fotografie von Renan an seinem Arbeitsplatz im Collège de France, ein Warenhaus von Steinberg, Cranachs Melanchthon – auf Holztafeln befestigt und in die Gefache eingelassen. Etwas links vom Fenster und leicht schräg stünde ein langer lothringischer, mit einem großen, roten Löschblatt bedeckter Tisch. Holzschalen, lange Federkästen, Behältnisse verschiedenster Art enthielten Bleistifte, Heftklammern, Karteikartenreiter. Ein Glasbaustein würde als Aschenbecher dienen. Eine runde Dose aus schwarzem Leder, mit feinen Goldarabesken verziert, wäre mit Zigaretten gefüllt. Das Licht käme von einer alten, schlecht verstellbaren Tischlampe mit einem Schirm in der Form eines Visiers aus grünem Opalglas. Zu beiden Seiten des Tisches, einander fast genau gegenüber, stünden zwei Sessel aus Holz und Leder mit hoher Rückenlehne. Noch weiter links, direkt an der Wand, würde ein schmaler Tisch von Büchern überquellen. Ein Klubsessel aus flaschengrünem Leder führte weiter zu graumetallenen Ablagekästen, zu Karteikästen aus hellem Holz. Auf einem dritten, noch kleineren Tisch ständen eine schwedische Lampe und eine mit einer Wachstuchhülle bedeckte Schreibmaschine. Ganz im Hintergrund gäbe es ein schmales, mit marineblauem Samt bezogenes Bett zu sehen, darauf Kissen in allen Farben. Ein Dreifuß aus bemaltem Holz trüge, fast in der Mitte des Zimmers, einen Globus aus Neusilber und Papiermachee, naiv bemalt, auf antik gemacht. Hinter dem Schreibtisch, halb verdeckt von dem roten Vorhang des Fensters, könnte auf einer Messingschiene eine Trittleiter aus gewachstem Holz, die rund um das Zimmer geführt werden würde, entlanggleiten.

Hier wäre das Leben leicht, wäre einfach. Alle Verpflichtungen und Probleme des Alltags fänden eine natürliche Lösung. Jeden Morgen wäre eine Zugehfrau da. Alle vierzehn Tage würden Wein, Öl, Zucker ins Haus geliefert. Es gäbe eine große, helle Küche mit wappengeschmückten, blauen Kacheln, drei arabeskenverzierten Wandtellern, überall metallisch glänzende Einbauschränke, einen schönen, weißen Holztisch in der Mitte, Hocker, Bänke. Es wäre angenehm, sich hier jeden Morgen nach dem Duschen leicht bekleidet hinzusetzen. Auf dem Tisch ständen eine Butterdose aus Steingut, Marmeladengläser, es gäbe Honig, Toast, halbierte Pampelmusen. Es wäre früh. Es wäre der Beginn eines langen Maitages.

Sie würden ihre Post öffnen, sie würden die Zeitungen aufschlagen, sie würden die erste Zigarette anzünden. Sie würden das Haus verlassen. Ihre Arbeit nähme sie nur ein paar Stunden am Vormittag in Anspruch. Zum Mittagessen träfen sie sich wieder, um, je nach Laune, ein Sandwich oder ein Stück gegrilltes Fleisch zu verzehren; auf einer Caféterrasse würden sie eine Tasse Kaffee trinken, danach würden sie zu Fuß langsam nach Hause gehen.

Ihre Wohnung wäre selten aufgeräumt, aber gerade die Unordnung wäre ihr größter Charme. Sie würden sich kaum um die Wohnung kümmern: Sie würden darin leben. Der Komfort, der sie umgäbe, würde ihnen selbstverständlich erscheinen, als eine feststehende Tatsache, als eine zu ihnen gehörende Gabe, als ein Zustand ihrer Natur. Ihre Aufmerksamkeit wäre anderen Dingen zugewandt: dem Buch, das sie aufschlügen, dem Text, den sie schrieben, der Schallplatte, die sie hörten, ihrem jeden Tag wieder neu begonnenen Dialog. Sie würden lange arbeiten, ohne Erregung, ohne Hast, ohne Groll. Dann würden sie zu Abend essen oder zum Abendessen ein Lokal aufsuchen; sie träfen ihre Freunde, sie gingen zusammen spazieren.

**Table A1:** Items of the Reading Experience Questionnaire Used in the Eye-tracking Experiment

No.	Items	English translation	Source
1	Beim Lesen hatte ich automatisch sensorische Vorstellungsbilder.	*Sensory images came to me automatically during reading.*	
2	Die Personen bzw. Orte wurden in meiner Vorstellung lebendig.	*The persons and places came to life in my imagination.*	based on Appel et al.’s [50] Imageability scale
3	Es war anstrengend, sich das Gelesene bildhaft vorszustellen.	*It was difficult to imagine visually what has been read.*	based on Appel et al.’s [50] Imageability scale
4	Ich war in der Lage, die Anordnungen der beschriebenen Objekte vor meinem inneren Auge zu sehen.	*I was able to see the arrangements of the described objects in my mind's eye*.	from Vorderer et al.’s [51] spatial presence questionnaire
5	Meine Gedanken sind beim Lesen immer wieder abgeschweift.	*While reading, my thoughts seemed to wander.*	from Appel et al.’s [50] Attentional focus scale
6	Ich habe die Welt um mich herum beim Lesen vergessen.	*While reading, I forgot the world around me.*	from Appel et al.’s [50] Immersion scale
7	Beim Lesen hatte ich das Gefühl, mich an einem anderen Ort zu befinden.	*While reading, I had the feeling that I was in a different place.*	from Appel et al.’s [50] Spatial presence scale
8	Ich war gespannt darauf zu erfahren, was wohl als nächstes passiert.	*I wanted to know how the events would unfold.*	from Appel et al.’s [50] Suspense scale
9	Der Text hat mich gefühlsmäßig berührt.	*The text affected me emotionally.*	from Appel et al.’s [50] Emotional Involvement scale
10	Was im Text beschrieben wurde, hat bei mir Emotionen ausgelöst.	*What was described in the text, made me feel emotions.*	based on Appel et al.’s [50] Emotional Involvement scale
11	Der Text hat mir gefallen.	*I liked the text.*	from Appel et al.’s [50] Overall Reading Pleasure scale
12	Ich habe beim Lesen wenig über die Inhalte nachgedacht.	*While reading I didn’t think much about the content of the text.*	from Appel et al.’s [50] Cognitive Involvement scale
13	Der Text hat mich gedanklich sehr beschäftigt.	*The text engaged me cognitively.*	from Appel et al.’s [50] Cognitive Involvement scale
14	Während des Lesens habe ich darauf geachtet, ob in dem Text alles zusammenpasst.	*While reading, I had the sense that everything in the text fit together.*	from Appel et al.’s [50] Coherence subscale (Analysis of reception scale)
15	Es fiel mir leicht den roten Faden zu finden.	*It was easy for me to find the thread of the story.*	from Appel et al.’s [50] Ease of cognitive access scale
16	Ich war beim Lesen unsicher, ob ich immer alles verstanden habe.	*During reading, I was not sure whether I had understood everything.*	from Appel et al.’s [50] Ease of cognitive access scale
17	Durch den Text habe ich eine Atmosphäre gespürt.	*I felt that the text conveyed an atmosphere.*	based on Lüdtke et al. [52]
18	Durch den Text konnte ich eine Situation nachempfinden.	*I could empathise about a situation with the help of the text.*	based on Lüdtke et al. [52]
